# Paediatric COVID-19 mortality: a database analysis of the impact of health resource disparity

**DOI:** 10.1136/bmjpo-2022-001657

**Published:** 2022-10-27

**Authors:** Eva Miranda Marwali, Aria Kekalih, Saptadi Yuliarto, Dyah Kanya Wati, Muhammad Rayhan, Ivy Cerelia Valerie, Hwa Jin Cho, Waasila Jassat, Lucille Blumberg, Maureen Masha, Calum Semple, Olivia V Swann, Malte Kohns Vasconcelos, Jolanta Popielska, Srinivas Murthy, Robert A Fowler, Anne-Marie Guerguerian, Anca Streinu-Cercel, Mohan Dass Pathmanathan, Amanda Rojek, Christiana Kartsonaki, Bronner P Gonçalves, Barbara Wanjiru Citarella, Laura Merson, Piero L Olliaro, Heidi Jean Dalton, International Severe Acute Respiratory and emerging Infection Consortium (ISARIC) Clinical Characterization Group Investigators, Sheryl Ann Abdukahil

**Affiliations:** 1 Department of Pediatric Cardiac Intensive Care, National Cardiovascular Center Harapan Kita, Jakarta, Indonesia; 2 International Severe Acute Respiratory and emerging Infection Consortium (ISARIC), Pandemic Sciences Institute, University of Oxford, Oxford, UK; 3 Department of Community Medicine, Faculty of Medicine, Universitas Indonesia, Jakarta, Indonesia; 4 Department of Pediatrics, Faculty of Medicine, Universitas Brawijaya, Saiful Anwar Hospital, Malang, Jawa Timur, Indonesia; 5 Department of Pediatrics, Faculty of Medicine, Universitas Udayana, Sanglah Hospital, Denpasar, Bali, Indonesia; 6 Division of Pediatric Cardiology and Pediatric Intensive Care, Department of Pediatrics, Chonnam National University Hospital, Gwangju, Korea (the Republic of); 7 National Institute for Communicable Diseases, Johannesburg, South Africa; 8 NIHR Health Protection Research Unit in Emerging and Zoonotic Infections, Institute of Infection Veterinary and Ecological Sciences, University of Liverpool, Liverpool, Merseyside, UK; 9 Centre for Medical Informatics, The University of Edinburgh Usher Institute of Population Health Sciences and Informatics, Edinburgh, UK; 10 Centre for Neonatal and Paediatric Infection, St George's University of London, London, UK; 11 Warsaw's Hospital for Infectious Diseases, Medical University of Warsaw, Warszawa, Mazowieckie, Poland; 12 Department of Pediatrics, Division of Critical Care, The University of British Columbia, Vancouver, British Columbia, Canada; 13 Interdepartmental Division of Critical Care Medicine, University of Toronto, Toronto, Ontario, Canada; 14 Department of Critical Care Medicine, Neurosciences and Mental Health Program, Faculty of Medicine, University of Toronto, The Hospital for Sick Children, Toronto, Ontario, Canada; 15 National Institute of Infectious Diseases Prof Dr Matei Bals, Bucuresti, Romania; 16 National Institutes of Health, Ministry of Health Malaysia, Putrajaya, Wilayah Persekutuan, Malaysia; 17 The Royal Melbourne Hospital, Parkville, Victoria, Australia; 18 Inova Fairfax Medical Center, Inova, Falls Church, Arizona, USA

**Keywords:** Mortality, Neonatology, Adolescent Health, Health services research, COVID-19

## Abstract

**Background:**

The impact of the COVID-19 pandemic on paediatric populations varied between high-income countries (HICs) versus low-income to middle-income countries (LMICs). We sought to investigate differences in paediatric clinical outcomes and identify factors contributing to disparity between countries.

**Methods:**

The International Severe Acute Respiratory and Emerging Infections Consortium (ISARIC) COVID-19 database was queried to include children under 19 years of age admitted to hospital from January 2020 to April 2021 with suspected or confirmed COVID-19 diagnosis. Univariate and multivariable analysis of contributing factors for mortality were assessed by country group (HICs vs LMICs) as defined by the World Bank criteria.

**Results:**

A total of 12 860 children (3819 from 21 HICs and 9041 from 15 LMICs) participated in this study. Of these, 8961 were laboratory-confirmed and 3899 suspected COVID-19 cases. About 52% of LMICs children were black, and more than 40% were infants and adolescent. Overall in-hospital mortality rate (95% CI) was 3.3% [=(3.0% to 3.6%), higher in LMICs than HICs (4.0% (3.6% to 4.4%) and 1.7% (1.3% to 2.1%), respectively). There were significant differences between country income groups in intervention profile, with higher use of antibiotics, antivirals, corticosteroids, prone positioning, high flow nasal cannula, non-invasive and invasive mechanical ventilation in HICs. Out of the 439 mechanically ventilated children, mortality occurred in 106 (24.1%) subjects, which was higher in LMICs than HICs (89 (43.6%) vs 17 (7.2%) respectively). Pre-existing infectious comorbidities (tuberculosis and HIV) and some complications (bacterial pneumonia, acute respiratory distress syndrome and myocarditis) were significantly higher in LMICs compared with HICs. On multivariable analysis, LMIC as country income group was associated with increased risk of mortality (adjusted HR 4.73 (3.16 to 7.10)).

**Conclusion:**

Mortality and morbidities were higher in LMICs than HICs, and it may be attributable to differences in patient demographics, complications and access to supportive and treatment modalities.

**WHAT IS ALREADY KNOWN ON THIS TOPIC**

- Recent systematic reviews identified a potentially higher paediatric mortality per population from COVID-19 in low-income to middle-income countries (LMICs) compared with high-income countries (HICs) but concluded that heterogeneity of published studies limits firm conclusions. Correspondingly, lethality of other acute respiratory infections has been shown to be higher in LMIC.

**WHAT THIS STUDY ADDS**

- By using harmonised data collection tools of a study population of over 12 000 children, this study can directly compare inpatient management and outcomes in HIC and LMIC. Analysis finds higher mortality in LMIC, although a lower proportion receive intensive care unit admission and ventilation prior to death. Disparity in access to care and lack of available advanced medical therapies are highlighted and provide areas for collaborative efforts between clinicians, administrators and likely government groups to improve outcomes in LMIC.

**HOW THIS STUDY MIGHT AFFECT RESEARCH, PRACTICE OR POLICY**

- While intensified by the pandemic, a lack of adequate resources to care for children with acute respiratory infections in LMIC is likely a general concern that requires allocation of resources. Reducing the gap in our ability to care for sick children in LMICs versus HICs will inevitably improve global outcomes during both pandemic and interpandemic periods.

## Introduction

The clinical presentation, severity and outcomes of acute COVID-19 are different in children compared with adults. While a higher proportion of children are asymptomatic or less severely ill than in many adult reports, severe manifestations do occur.^1^ While cardiac compromise in the form of multisystem inflammatory syndrome in children (MIS-C) is often described, acute respiratory distress syndrome (ARDS) and other organ dysfunction also occurs in children.^2 3^ The risk factors for severe disease in in paediatrics are incompletely understood.^4^ Furthermore, there is a lack of global data to improve understanding of the COVID-19 burden in children who live in low-income to middle-income countries (LMICs) versus those in high-income countries (HICs) sites.^5^

One systematic review that summarised the difference in paediatric COVID-19 morbidity and mortality in HICs and LMICs has been published.^6^ However, most studies’ samples analysed fewer than 100 patients, and over half the data came from the USA and China. Furthermore, due to heterogeneous reporting of data in the included studies, the authors were limited in their ability to pool or compare data on clinical features and outcomes. A recent scoping review reported that ethnicity and socioeconomic condition were under-represented in COVID-19 epidemiological studies.^7^ We aimed to investigate the differences in survival of paediatric patients in LMICs and HICs and the factors that may contribute to such differences between regions.

## Methods

Data were collected from hospitalised patients under 19 years of age with confirmed or clinically suspected COVID-19 between 1 January 2020 and 31 March 2021 admitted to institutions across the globe contributing to the International Severe Acute Respiratory and Emerging Infections Consortium (ISARIC) database according to ISARIC/WHO Clinical Characterisation Protocol for Severe Emerging Infection.^1 8^ Fields for analysis were extracted from the complete dataset gathered from Research Electronic Data Capture (V.8.11.11, Vanderbilt University, Nashville, Tennessee, USA).^8^

Variables of interest were classified into four domains: comorbidities, presenting signs and symptoms, complications and treatments. Comorbidity was defined as any history of pre-existing medical conditions that were not otherwise related to COVID-19 natural history and reported at admission date. Complications referred to any medical condition detected during patients’ stay that was not present at admission. Therapies included were drugs, oxygen and use of other treatments such as invasive mechanical ventilation (IMV). Ethnicity was collapsed into five categories (black or African American, white, Asian, mixed/others and missing/unknown) following the Centers for Disease Control and Prevention National Health Interview Survey glossary.^9^ Country income groups were dichotomised into HICs and LMICs according to the latest World Bank classification.^10^

Date of admission was defined as the date of hospitalisation. Descriptive statistics were described as frequencies (n) and proportions for categorical data, mean±SD or median (IQR) for continuous data, and number of available data (N) for each variable. Demographic characteristics, comorbidities, complications and treatments were compared between country income groups using χ^2^ or Fisher’s exact test as indicated. Kaplan-Meier survival curves were plotted and compared using the log-rank test. Multivariable Cox proportional hazards regression models were fitted to identify mortality predictors. In-hospital survival analysis was performed to obtain 28-day and 90-day survival rates. Intensive care unit (ICU) admission and IMV requirement served as proxies for morbidity. Time-to-event analyses were performed to identify morbidity and mortality defining periods: (1) from hospital admission until ICU admission, (2) ICU admission to first intubation, (3) ICU admission to ICU discharge or death, (4) ICU discharge to hospital discharge or death. A p value of <0.05 was considered statistically significant. Statistical analyses were performed using SPSS V.25 (IBM Corp).

## Results

**Figure 1:**
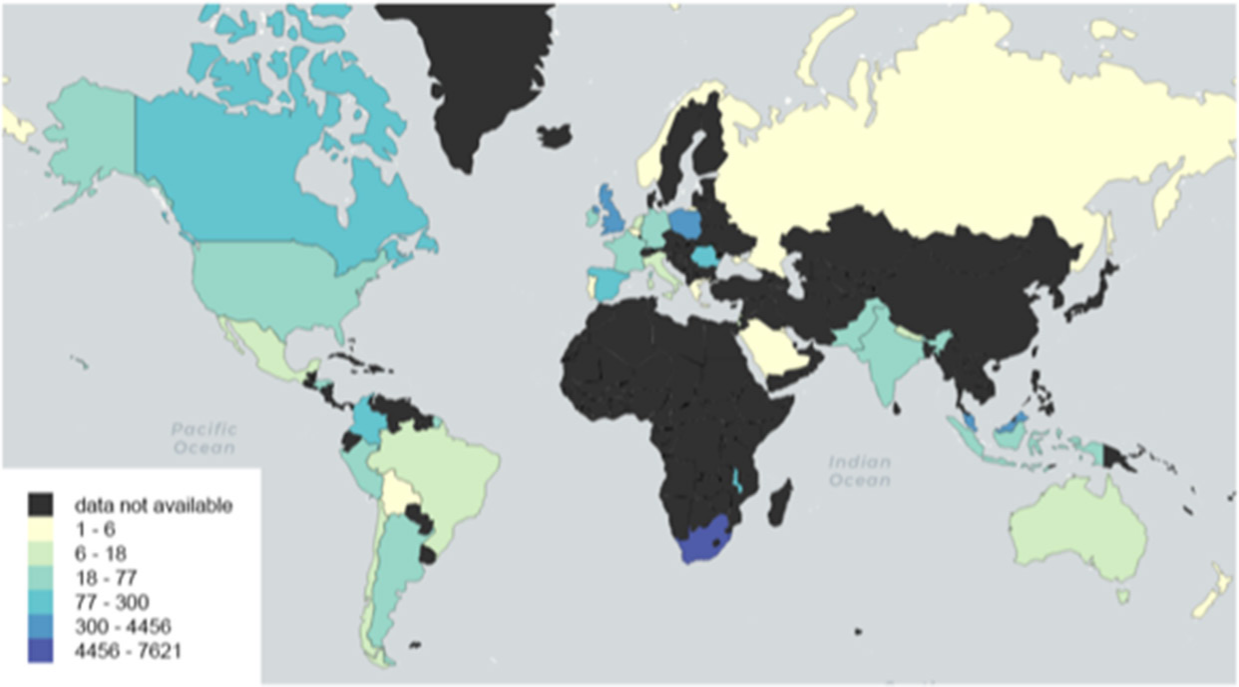
Distribution of participants by country of origin.

There were 12 860 children, originating from 36 countries, 15 of which (41.7%) were LMICs and 21 (58.3%) HICs(table 1). Seventy per cent (n=9041) of participants were from LMICs and 29.7% (n=3819) cases were from HICs. The majority of participants were contributed from 670 cites in South Africa (59.3%) and 346 sites in the UK (24.1%), followed by Malaysia (4.9%), Poland (2,4%) and Malawi (2.3%) (figure 1 and table 1). COVID-19 status was laboratory confirmed in 69.7%; this rate was higher in HICs (73.0%) in comparison with that in LMICs (68.3%) (table 2).

**Table 1:** List of participating countries and number of admitted subjects (n=12 860)

Income group	Country name	n	%
High-income country	UK	3099	24.1
High-income country	Poland	304	2.4
High-income country	Canada	164	1.3
High-income country	Spain	87	0.7
High-income country	Germany	25	0.2
High-income country	USA	22	0.2
High-income country	Ireland	21	0.2
High-income country	France	18	0.1
High-income country	Australia	17	0.1
High-income country	Chile	16	0.1
High-income country	Israel	12	0.1
High-income country	Netherlands	11	0.1
High-income country	Italy	8	0.1
High-income country	Greece	4	0.0
High-income country	Belgium	3	0.0
High-income country	Portugal	3	0.0
High-income country	Bolivia	1	0.0
High-income country	Kuwait	1	0.0
High-income country	Norway	1	0.0
High-income country	New Zealand	1	0.0
High-income country	Saudi Arabia	1	0.0
Lower-middle income country	South Africa	7621	59.3
Lower-middle income country	Malaysia	627	4.9
Lower-middle income country	Malawi	296	2.3
Lower-middle income country	Romania	135	1.0
Lower-middle income country	Colombia	112	0.9
Lower-middle income country	Indonesia	73	0.6
Lower-middle income country	Peru	49	0.4
Lower-middle income country	Pakistan	35	0.3
Lower-middle income country	India	24	0.2
Lower-middle income country	Honduras	21	0.2
Lower-middle income country	Argentina	18	0.1
Lower-middle income country	Mexico	11	0.1
Lower-middle income country	Brazil	10	0.1
Lower-middle income country	Nepal	6	0.0
Lower-middle income country	Russia	3	0.0

**Table 2:** Baseline characteristics of all study participants (n=12 860)

		Country income						P value
		HICs (n=3819)		LMICs (n=9041)		Total		
		n	%	n	%	n	%	
Age*	<1 year	1124	29.4	1574	17.4	2698	21.0	<0.001
	1–<5 years	707	18.5	1887	20.9	2594	20.2	
	5–<12 years	796	20.8	1621	17.9	2417	18.8	
	12–<17 years	770	20.2	2311	25.6	3081	24.0	
	≥17 years	422	11.1	1648	18.2	2070	16.1	
Sex*	Male	2026	53.1	4497	49.8	6523	50.8	0.002
	Female	1788	46.9	4537	50.2	6325	49.2	
Ethnicity*	Black or African-American	223	5.8	4747	52.5	4970	38.6	<0.001
	Missing and unknown	1221	32.0	3509	38.8	4730	36.8	
	White	1624	42.5	186	2.1	1810	14.1	
	Asian	561	14.7	168	1.9	729	5.7	
	Mixed and others	190	5.0	431	4.8	621	4.8	
COVID-19 status*	Confirmed	2786	73	6175	68.3	8961	69.7	<0.001
	Suspect	1033	27	2866	31.7	3899	30.3	
Outcome*	Discharged	3423	89.6	7967	88.1	11 390	88.6	<0.001
	Still in hospital	140	3.7	357	3.9	497	3.9	
	Transferred	192	5.0	356	3.9	548	4.3	
	Death	64	1.7	361	4.0	425	3.3	

*Significant with p value <0.05 using χ^2^ test.

HICs, high-income countries; LMICs, low- to middle-income countries.

Adolescents aged between 12 and 17 years were the largest age group in LMICs (25.6%) and overall (24.0%), followed by infants younger than 1 year of age (21%). Infants were the largest group in HICs (29.4%). Males (50.8%) and females (49.2%) were similarly represented. Black or African-American participants formed more than one-third of the study population and 52% of LMICs children (table 2).

**Figure 2:**
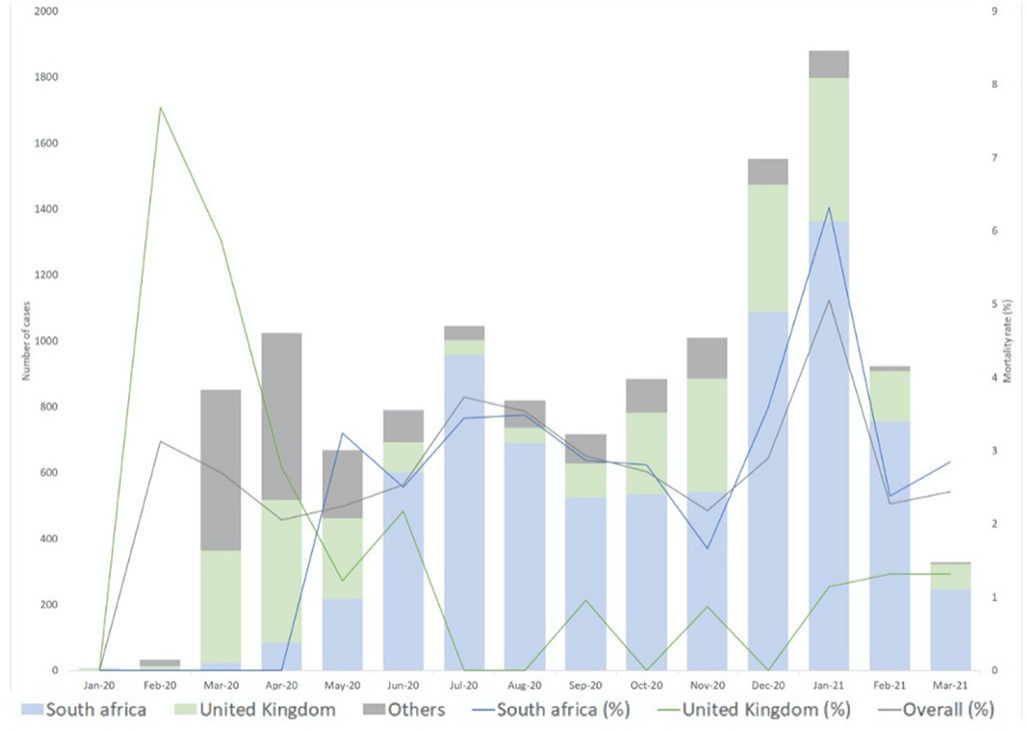
Total COVID-19 participants (bar) and mortality (line) rate trend through out data collection period.

In total, 425 (3.3%) participants died. The mortality rate was higher in in LMICs compared with HICs (4% vs 1.7%) (table 2). Reported mortality in the UK was 52 children (1.2%), which does not differ significantly (p=0.98) from the rest of HICs. Mortality in South Africa, numbering 268 children (3.5%), differed significantly (p<0.001) from 93 (6.5%) mortality in the rest of LMICs. The total admissions and mortality rates across the study period based on country groups (UK, South Africa and other countries) were presented in figure 2. The curves showed bimodal with peaks of mortality rates coinciding with admissions during July 2020 and January 2021. Available case data in early 2020 was limited compared with later time points. In contrast to South Africa and other countries, mortality rate in the UK did not rise significantly with the first and second wave of case admissions during April–May 2020 and October 2020–January 2021.

Children in LMICs had significantly greater and earlier mortality (adjusted HR (aHR) (95% CI) 4.73 (3.16 to 7.10), p<0.001). The 28-day and 90-days survival among all participants were 96.7% (10 339/10 692) and 96.5% (10 642/11 024), respectively. Survival was higher at both 28 (98.3% (3049/3101)) and 90 days (98.1% (3137/3199)) in HICs than LMICs (96.0% (7290/7591) and 95% (7505/7825), respectively).

The availability of information on comorbidities varied between countries of origin. The prevalence of several comorbidities was significantly higher in HICs including chronic neurological disease, seizures, diabetes and chronic cardiac disease. Infectious diseases such as tuberculosis and HIV/AIDS were significantly higher in LMICs (table 3).

**Table 3:** Comparison of comorbidities between country income groups

Country income										
	HICs			LMICs			Total			P value
	N	n	%	N	n	%	N	n	%	
Chronic neurological disorder*	2588	191	7.4	902	14	1.6	3490	205	5.9	0.001
Seizure*	2490	111	4.5	899	16	1.8	3389	127	3.7	0.001
Smoking	2091	82	3.9	1509	46	3.0	3600	128	3.6	0.215
Diabetes*	2592	95	3.7	4468	149	3.3	7060	244	3.5	0.001
Chronic cardiac disease*	2587	141	5.5	3578	48	1.3	6165	189	3.1	0.001
Obesity*	2438	90	3.7	1736	39	2.2	4174	129	3.1	0.001
Tuberculosis*	290	–	–	3649	102	2.8	3939	102	2.6	0.001
Chronic haematological disease*	2475	77	3.1	899	9	1.0	3374	86	2.5	0.001
HIV/AIDS*	2562	1	<0.01	4382	142	3.2	6944	143	2.1	0.001
Rare diseases and inborn errors of metabolism	1823	36	2.0	0	–	–	1823	36	2.0%	0.715
Malnutrition	2539	35	1.4	901	26	2.9%	3440	61	1.8%	0.412
Malignant neoplasm*	2571	64	2.5	4164	27	0.6%	6735	91	1.4%	0.001
Rheumatological disorder*	2491	45	1.8	898	2	0.2%	3389	47	1.4%	0.001
Chronic kidney disease*	2596	59	2.3	4147	28	0.7	6743	87	1.3	0.001
Liver disease	2623	12	0.5	902	–	–	3525	12	0.3	0.866

*Significant with p-value <0.05 using Chi-square test.

HICs, high-income countries; LMICs, low-income to middle-income countries; N, denominators; n, total patients with comorbidities.

The most common presenting symptoms overall include fever (20%), cough (16.1%) and shortness of breath(10.5%). Between-group’s comparisons showed that cough (HIC 14.0% vs LMIC 26.1%), shortness of breath (9.2% vs 16,4%), runny nose (4.2% vs 8.6%), loss of smell or taste (0.7% vs 2.9%), anorexia (0.1% vs 0.8%) and inability to walk (0.1% vs 0.2%) were more common among patients in LMICs.

Complications during hospitalisation are shown in table 4. Patients in LMICs had more bacterial and cryptogenic pneumonia and ARDS, as well as other organ involvement such as brain with stroke or heart with cardiac arrest. The rates of complications were generally higher than that of HICs, except for cardiac arrhythmia and disseminated intravascular coagulation in which HICs rates were statistically higher.

**Table 4:** Comparison of complications between country income groups

Country income										
	HICs			LMICs			Total			P value
	N	n	%	N	n	%	N	n	%	
Bacterial pneumonia*	3497	11 5	3.3	1107	10 2	9.2	4604	21 7	4.7	<0.001
ARDS*	3153	31	1.0	4972	26 7	5.4	8125	29 8	3.7	<0.001
AKI	3624	11 0	3.0	1410	49	3.5	5034	15 9	3.2	0.477
Seizure	3627	10 3	2.8	1412	39	2.8	5039	14 2	2.8	0.958
Pleural effusion	3511	82	2.3	1110	36	3.2	4621	11 8	2.6	0.118
Myocarditis and pericarditis*	2858	42	1.5	427	22	5.2	3285	64	1.9	<0.001
Bronchiolitis	3620	57	1.6	1412	21	1.5	5032	78	1.6	0.922
Cardiac arrhythmia*	3635	70	1.9	1407	13	0.9	5042	83	1.6	0.017
Endocarditis*	692	5	0.7	487	14	2.9	1179	19	1.6	0.008
Cardiac arrest*	3535	19	0.5	1412	55	3.9	4947	74	1.5	<0.001
DIC*	3498	48	1.3	4668	58	1.2	8166	10 4	1.3	0.850
Meningitis and encephalitis*	3621	16	0.4	1413	20	1.4	5034	36	0.7	<0.001
Pneumothorax	3530	15	0.4	1109	9	0.8	4639	24	0.5	0.185
Stroke/cerebrovascular complication†	3527	7	0.2	1109	11	1.0	4636	18	0.4	0.001
Pulmonary embolism	1928	4	0.2	293	2	0.7	2221	6	0.3	0.182
Cardiac ischaemia	3510	7	0.2	1110	4	0.4	4620	11	0.2	0.309
Myocardial infarction	312	1	0.3	298	–	–	610	1	0.2	1.000
Sepsis	–	–	–	3862	8	0.2	3862	8	0.2	–
DVT	1606	1	0.1	3	–	–	1609	1	0.1	1.000
COP†	3398	–	–	1110	5	0.5	4508	5	0.1	0.001

*Significant with p-value <0.05 using χ^2^ test.

†Fisher’s exact test.

AKI, acute kidney injury; ARDS, acute respiratory distress syndrome; COP, cryptogenic organising pneumonia; DIC, disseminated intravascular coagulation; DVT, deep vein thrombosis; HICs, high-income countries; LMICs, low-income to middle-income countries; n, total patients with complications; N, denominators.

The two most commonly administered therapies were antibiotics in 41.2% of participants, followed by corticosteroids in 11.4%. Antivirus was administered in 5.3% of subjects, with higher percentage observed in HIC, especially of remdesivir, which is more than twice that of LMIC. Adjunctive and supportive treatments were generally performed more often in HICs. No participants in LMICs were treated with extracorporeal membrane oxygenation, as compared with 10 participants in HICs (table 5).

**Table 5:** Treatment profile of study participants

	Country income									P value
	HICs			LMICs			Total			
	N	n	%	N	n	%	N	n	%	
Antibiotics*	3685	2189	59.4	4349	1122	25.8	8034	3311	41.2	<0.001
Corticosteroid*	3663	564	15.4	4905	417	8.5	8568	981	11.4	<0.001
HFNC*	3380	282	8.3	3967	147	3.7	7347	429	5.8	<0.001
IMV*	3620	235	6.5	4684	204	4.3	8304	439	5.3	<0.001
Antivirus*	3672	271	7.4	4341	172	4.0	8013	443	5.3	<0.001
Remdesivir	3672	96	2.6	4341	17	0.4	8013	113	1.4	
Neuraminidase inhibitor	3672	32	0.9	4341	23	0.5	8013	113	55	
Inotropic/vasopressor*	3451	201	5.8	4961	174	3.5	8412	375	4.4	<0.001
Prone positioning*	3532	64	1.8	4647	39	0.8	8179	103	1.2	<0.001
Anticoagulant	3819	50	1.3	9041	88	1.0	12 860	138	1.1	0.091
NIV*	3627	53	1.5	4966	17	0.3	8593	70	0.8	<0.001
RRT	3573	19	0.5	4334	27	0.6	7907	46	0.6	0.084
ECMO†	3608	10	0.3	1086	–	–	4694	10	0.2	<0.001

*Significant with p value <0.05 using χ^2^ test.

†Fisher’s exact test.

ECMO, extracorporeal membrane oxygenation; HFNC, high-flow nasal cannula; HICs, high-income countries; IMV, invasive mechanical ventilation; LMICs, low-income to middle-income countries; N, denominators; n, total patients with treatments; NIV, non-invasive ventilation; RRT, renal replacement therapy.

Participants in LMICs were most often admitted to the ICU within the first day of admission, with those who died being admitted earlier than survivors (LMICs vs HICs: 0 (0–1) vs 0 (0–2.5) day respectively, p=0.03). While time to IMV was not significantly different for survivors versus non-survivors in either LMIC or HICs, most received IMV in the first day of admission and all within a maximum of 3.5 days. Time from ICU admission to ICU discharge or death was significantly shorter in survivors versus non-survivors, although non survivors in HICs had longer stays than in LMICs (3 (1–6) vs 9.5 (4.5–19.2) days respectively in HIC and 0 (0–4) vs 4 (0.7–7.2) days respectively in LMIC, p<0.05). Mortality was significantly higher (p<0.001) in LMICs compared with HICs for participants who received IMV (43.6% vs 7.3%) or who required ICU admission (16.7% vs 3.5). Nearly 2.3% and 6.1% participants died without ICU and/or IMV support in LMICs in comparison with the respective 1.3% and 1.4% in HICs (table 6).

**Table 6:** Mortality based on ICU admission and IMV use

Mortality based on country income										
		HICs			LMICs			Total		
		N	n	%	N	n	%	N	n	%
ICU*	Yes	679	24	3.5	1037	173	16.7	1716	197	11.5
	No	3140	40	1.3	8004	188	2.3	11 144	228	2.0
IMV*	Yes	235	17	7.2	204	89	43.6	439	106	24.1
	No	3385	47	1.4	4480	272	6.1	7865	319	4.0

*Significant with p value <0.05 using χ^2^ test.

HICs, high-income countries; ICU, intensive care unit; IMV, invasive mechanical ventilation; LMICs, low-income to middle-income countries; N, denominator (total patients with or without ICU/ IMV); n, total mortality.

**Figure 3:**
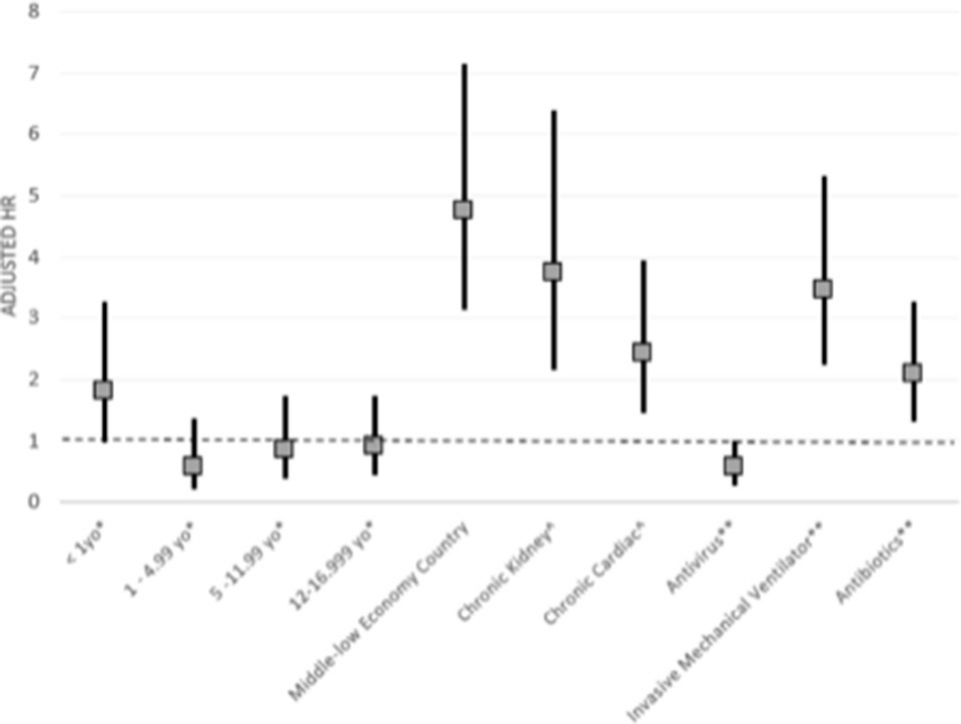
Adjusted HR and 95% CI of mortality risk factors in all participants.

Multivariable analysis was done to evaluate factors associated with mortality. Significant risk factors found were: aged <1 year (aHR (95% CI)=1.80 (1.01 to 3.22)), low-middle income group (4.73 (3.16 to 7.10)), comorbidities such as chronic kidney disease (3.74 (2.20 to 6.35)) or cardiac disease (2.42 (1.50 to 3.91)) and invasive mechanical ventilator requirement (3.46 (2.27 to 5.28)) or exposure to antibiotics (2.07 (1.34 to 3.22)). The use of antiviral agents (aHR=0.55 (0.32 to 0.96)) was the only factor inversely associated with mortality (figure 3).

## Discussion

We present a large international cohort of children hospitalised with COVID- 19. We found that mortality was significantly higher in LMICs in comparison with HICs. Disparate care patterns were also observed, with patients in LMICs reported to receive most adjunctive and supportive therapies less frequently than patients in HICs. While these findings may represent differences in practice, they may also represent variation in available supports for children based on income status of the country. Such disparities have been described in adult COVID-19 patients, but limited data exist for children. Prior reports have focused on specific aspects of illness such as infection or cardiac dysfunction, have included small cohorts of children or are limited to certain countries or regions.^11–20^

While the findings may be criticised as mainly representing data from two countries, the UK and South Africa (SA), these countries are good examples of HICs and LMICs. Statistical analysis showed no significant difference of mortality between UK and the rest of HICs. Although mortality in SA was significantly lower than the rest of LMICs, both mortalities from SA and non-SA LMICs were significantly higher than HICs group. Low number of subjects from non-SA LMICs was disproportionate to that of SA, thus conclusion can not be drawn from observed difference in mortality between them. Furthermore, as data supplied from the UK and South Africa comes from national COVID-19 research databases recruiting from a high number of sites in the UK and South Africa, and in this sense may be more representative of country income differences, as opposed to enrolment of single sites (eg, a national referral centre) in different countries. The inclusion of children from many other countries, although relatively small cohorts form each country in comparison, does allow understanding of care patterns in areas around the world.

More participants from HICs could be admitted to ICU and received IMV than LMICs patients. While the small numbers available for analysis in some categories limit our confidence in these findings, in LMICs they do given IMV and dying within shorter periods of time than HICs. Not only were children in LMICs hospitalised with COVID-19 more likely to die, they were also shown to die earlier in their hospitalisation. Despite possible confounding effects from missing data relating to severity of illness at presentation (vital signs, organ failure scores), a positive association between LMICs and mortality were consistently observed in analysis of children admitted to the ICU and those receiving IMV.

Several independent risk factors for mortality were identified in addition to country economic group. Mortality was lowest for patients aged between 1 and 5 years and higher among patients of age <1 or >5 years. This finding confirmed the U-shaped mortality pattern shown in several other reports, although infancy is not always recognised as a risk factor in small studies.^1 21 22^ Comorbidities such as chronic kidney and cardiac diseases were also shown to be independent risk factors as reported by others.^19 23 24^ These risk factors were more prevalent in HICs and thus did not seem to be associated with the high mortality rates noted in LMICs, but this may reflect underdiagnosis possibly due to lack of diagnostic resources in some LMICs.^20^ Higher rates of certain comorbidities, many infectious in nature, is another possible cause of the relationship between LMICs and mortality. Chronic respiratory failure has been associated with death in COVID-19 adult patients, with some evidence that this is occurs in paediatric cases as well. Our data also provided information on tuberculosis, which has not specifically identified as comorbidity in children with COVID-19 in other reports. Similarly, data on the impact of HIV in children is sparse, and our review finds this to be an important risk factor and more prevalent in LMICs.^23 25^

More patients receiving antiviral therapy were found in HICs versus LMICS. In fact, remdesivir, which is recommended for severe hospitalised COVID-19, was used in exceptionally lower percentage of LMIC subjects. We can only speculate that period of this study occurred when evidence based on antiviral efficacy was still scarce especially in children, or it may indicate lack of access to drug or lack familiarity with recommendations.^26^ The recommendations for antiviral use in children with severe COVID-19 from the National Institutes of Health have suggested use for patients over the age of 12 years; this recommendation would not have applied to infants who had a higher risk of mortality in our study.^27 28^ Further investigation of the impact of antivirals in children of all ages should be considered. The efficacy and the cost benefit of these expensive medications in resource-limited sites are needed; if valuable, improving access should then be at the core of discussions.

The use of other therapies also highlight the differences between LMICs and HICs. There was less use of many adjunct therapies associated with outcomes in adult studies such as prone positioning, high flow nasal cannula, antibiotics and steroids in LMIC sites. Whether these differences were the result of lack of availability of therapies or other regional factors cannot be determined, but it seems likely that limitation to access may influence practice. The high rate of mortality in patients outside the ICU and who died without IMV suggests that limitations to ICU beds or ventilators in LMICs likely play a large role in the excess death rates reported as compared with HICs.^29 30^

The higher prevalence of complications of respiratory disease such as ARDS, bacterial and cryptogenic organising pneumonia, and the impact of organ dysfunction outside the lung such as increased rates of myocarditis, pericarditis, endocarditis, meningitis, encephalitis, stroke and cardiac arrest observed in LMICs are also likely factors in the high death rates. Patients with MIS-C were not specifically reported in the time period of this report.

Our study has the strength of a common reporting format in participating centres around the world. We describe a relatively large number of children and are able to provide both comparisons of patient characteristics and outcomes and evaluate risk factors for outcomes using common definitions. Limitation of this study includes a predominance of patients from one LMICs and one HICs, South Africa and UK, potentially limiting the generalisability of our findings to all countries. In addition, we did not adjust for pandemic era. Inevitably, we have missing data for a number of variables, including comorbidities, which limits the effective sample size of analyses examining relationships with patient characteristics and outcomes. Lack of data on nutritional status of children on each group, which may explain disparity between country income groups, was another limitation of the study. Moreover, considerable proportion of non-confirmed cases also limits the impact of this study on public health policy.

In conclusion, we found many differences in characteristics, treatments and outcomes among children from LMICs and HICs with infants had higher death rates than other children. Patients less frequently receive IMV and other supportive therapies in LMICs, which likely represents disparities in access to healthcare that influence outcomes. Reducing the gap in our ability to care for sick children in LMICs versus HICs will inevitably improve global outcomes during both pandemic and interpandemic periods.

## Supplementary Material

Reviewer comments

Author's manuscript

## Data Availability

Data are available on reasonable request.
