# Supplementary material for: Paediatric COVID-19 mortality: a database analysis of the impact of health resource disparity
Source: BMJ Paediatr Open. 2022 Oct 27;6(1):e001657. doi: 10.1136/bmjpo-2022-001657 (PMC9621167; doi:10.1136/bmjpo-2022-001657)
Supplement: Author's manuscript [file bmjpo-2022-001657.draft_revisions.pdf]

BMJ Paediatrics Open

BMJ Paediatrics Open is committed to open peer review. As part of this commitment we make the peer review history of every article we publish publicly available.

When an article is published we post the peer reviewers' comments and the authors' responses online. We also post the versions of the paper that were used during peer review. These are the versions that the peer review comments apply to.

The versions of the paper that follow are the versions that were submitted during the peer review process. They are not the versions of record or the final published versions. They should not be cited or distributed as the published version of this manuscript.

BMJ Paediatrics Open is an open access journal and the full, final, typeset and author-corrected version of record of the manuscript is available on our site with no access controls, subscription charges or pay-per-view fees (http://bmjpaedsopen.bmj.com).

If you have any questions on BMJ Paediatrics Open's open peer review process please email info.bmjpo@bmj.com

BMJ Paediatrics Open

PEDIATRIC COVID-19 MORTALITY: TWO-SIDED CHALLENGES FROM HEALTH RESOURCE DISPARITY

Journal:	*BMJ Paediatrics Open*
Manuscript ID	bmjpo-2022-001657
Article Type:	Original research
Date Submitted by the Author:	26-Aug-2022

Complete List of Authors:	Marwali, Eva; National Cardiovascular Center, Pediatric Cardiac Intensive Care, National Cardiovascular Center Harapan Kita<br>Kekalih, Aria; Universitas Indonesia Fakultas Kedokteran, Community Medicine Department<br>Yuliarto, Saptadi; Brawijaya University Faculty of Medicine, Department of Pediatrics; Rumah Sakit Umum Dr Saiful Anwar, Saiful Anwar Hospital, Malang, Indonesia<br>Wati, Dyah K.; Udayana University Faculty of Medicine, Department of Child Health<br>Rayhan, Muhammad; National Cardiovascular Center, Pediatric Cardiac ICU, National Cardiovascular Center Harapan Kita, Jakarta, Indonesia<br>Valerie, Ivy C.; Udayana University Faculty of Medicine, Department of Child Health<br>Cho, Hwa; Chonnam National University Hospital, Division of Pediatric Cardiology and Pediatric Intensive Care, Department of Pediatrics, Chonnam National University Children's Hospital, Gwangju, South Korea<br>Jassat, Waasila; National Institute for Communicable Diseases<br>Blumberg, Lucille; National Institute for Communicable Diseases<br>Masha, Maureen; National Institute for Communicable Diseases<br>Semple, Calum; University of Liverpool, NIHR Health Protection Research Unit in Emerging and Zoonotic Infections, Institute of Infection Veterinary and Ecological Sciences, University of Liverpool, UK; University of Oxford, International Severe Acute Respiratory and emerging Infection Consortium (ISARIC), Pandemic Sciences Centre, University of Oxford, UK<br>Swann, Olivia; The University of Edinburgh Usher Institute of Population Health Sciences and Informatics, Centre for Medical Informatics<br>Vasconcelos, Malte Kohns; St George's University of London, Centre for Neonatal and Paediatric Infection<br>Popielska, Jolanta; Medical University of Warsaw; Warsaw's Hospital for Infectious Diseases<br>Murthy, Srinivas; The University of British Columbia, Department of Pediatrics, Division of Critical Care<br>Fowler, Robert; University of Toronto, Interdepartmental Division of Critical Care Medicine<br>Guerguerian, Anne-Marie ; The Hospital for Sick Children Department of Critical Care Medicine, Department of Critical Care Medicine, Neurosciences and Mental Health Program; University of Toronto Faculty of Medicine, Department of Critical Care Medicine

	Streinu-Cercel, Anca; National Institute of Infectious Diseases Prof Dr Matei Bals<br>Pathmanathan, Mohan D.; Ministry of Health Malaysia, National Institutes of Health<br>Rojek, Amanda; University of Oxford, International Severe Acute Respiratory and emerging Infection Consortium (ISARIC), Pandemic Sciences Centre; The Royal Melbourne Hospital<br>Kartsonaki, Christiana; University of Oxford, International Severe Acute Respiratory and emerging Infection Consortium (ISARIC), Pandemic Sciences Centre<br>Gonçalves, Bronner P.; University of Oxford, International Severe Acute Respiratory and emerging Infection Consortium (ISARIC), Pandemic Sciences Centre<br>Citarella, Barbara; University of Oxford, International Severe Acute Respiratory and emerging Infection Consortium (ISARIC), Pandemic Sciences Centre<br>Merson, Laura; University of Oxford, International Severe Acute Respiratory and emerging Infection Consortium (ISARIC), Pandemic Sciences Centre<br>Olliaro, Piero; University of Oxford, International Severe Acute Respiratory and emerging Infection Consortium (ISARIC), Pandemic Sciences Centre<br>Dalton, Heidi; Inova, Inova Fairfax Medical Center
Keywords:	Mortality, Neonatology, Adolescent Health, Health services research, COVID-19

SCHOLARONE™
Manuscripts

I, the Submitting Author has the right to grant and does grant on behalf of all authors of the Work (as defined in the below author licence), an exclusive licence and/or a non-exclusive licence for contributions from authors who are: i) UK Crown employees; ii) where BMJ has agreed a CC-BY licence shall apply, and/or iii) in accordance with the terms applicable for US Federal Government officers or employees acting as part of their official duties; on a worldwide, perpetual, irrevocable, royalty-free basis to BMJ Publishing Group Ltd ("BMJ") its licensees and where the relevant Journal is co-owned by BMJ to the co-owners of the Journal, to publish the Work in this journal and any other BMJ products and to exploit all rights, as set out in our licence.

The Submitting Author accepts and understands that any supply made under these terms is made by BMJ to the Submitting Author unless you are acting as an employee on behalf of your employer or a postgraduate student of an affiliated institution which is paying any applicable article publishing charge ("APC") for Open Access articles. Where the Submitting Author wishes to make the Work available on an Open Access basis (and intends to pay the relevant APC), the terms of reuse of such Open Access shall be governed by a Creative Commons licence – details of these licences and which Creative Commons licence will apply to this Work are set out in our licence referred to above.

Other than as permitted in any relevant BMJ Author's Self Archiving Policies, I confirm this Work has not been accepted for publication elsewhere, is not being considered for publication elsewhere and does not duplicate material already published. I confirm all authors consent to publication of this Work and authorise the granting of this licence.

Confidential: For Review Only

PEDIATRIC COVID-19 MORTALITY: TWO-SIDED CHALLENGES FROM HEALTH RESOURCE DISPARITY

First author: Eva M. Marwali[1*]

Co-authors: Aria Kekalih[2], Saptadi Yuliarto[3], Dyah K. Wati[4], Muhammad Rayhan[1], Ivy C. Valerie[4], Hwa J. Cho[5], Waasila Jassat[6], Lucille Blumberg[6], Maureen Masha[6], Malcolm G. Semple[7,8] , Olivia V. Swann[9], Malte Kohns Vasconcelos [10], Jolanta Popielska[11], Srinivas Murthy[12], Rob Fowler[13], Anne Marie Guerguerian[14], Anca Streinu-Cercel[15], Mohan D. Pathmanathan[16], Amanda Rojek[8,17], Christiana Kartsonaki[8], Bronner P. Gonçalves[8], Barbara Citarella[8], Laura Merson[8], Piero L. Olliaro[8], Heidi Dalton[18] and on behalf of International Severe Acute Respiratory and emerging Infection Consortium (ISARIC) Clinical Characterisation Group Investigators.

Affiliation:

[1] Pediatric Cardiac ICU, National Cardiovascular Center Harapan Kita, Jakarta, Indonesia

[2] Community Medicine Department, Faculty of Medicine, Universitas Indonesia, Jakarta, Indonesia

[3] Department of Pediatrics, Faculty of Medicine, Universitas Brawijaya, Saiful Anwar Hospital, Malang, Indonesia

[4] Department of Child Health, Faculty of Medicine, Universitas Udayana/Sanglah General Hospital, Bali, Indonesia

[5] Division of Pediatric Cardiology and Pediatric Intensive Care, Department of Pediatrics, Chonnam National University Children's Hospital, Gwangju, South Korea

[6] National Institute for Communicable Diseases, Johannesburg, South Africa; Right to Care, Pretoria, South Africa

[7] NIHR Health Protection Research Unit in Emerging and Zoonotic Infections, Institute of Infection Veterinary and Ecological Sciences, University of Liverpool, UK

[8] International Severe Acute Respiratory and emerging Infection Consortium (ISARIC), Pandemic Sciences Centre, University of Oxford, UK

[9] Centre for Medical Informatics, Usher Institute, University of Edinburgh, UK

[10] Centre for Neonatal and Paediatric Infection, St. Georges, University of London, London, UK

[11] Medical University of Warsaw and Hospital for Infectious Diseases in Warsaw, Poland

Confidential: For Review Only

[12] Division of Critical Care, Department of Pediatrics, University of British Columbia, Vancouver, Canada

[13] Interdepartmental Division of Critical Care Medicine, University of Toronto, Canada

[14] Department of Critical Care Medicine, Neurosciences and Mental Health Program, The Hospital for Sick Children, University of Toronto, Toronto, Canada.

[15] National Institute for Infectious Diseases Matei Bals, Romania

[16] Institute for Clinical Research, National Institutes of Health, Ministry of Health, Malaysia

[17] Royal Melbourne Hospital, Melbourne, Australia

[18] Inova Fairfax Medical Center, Fall Church, Arizona, USA

Corresponding author:

Eva M. Marwali, MD, PhD

Pediatric Cardiac ICU, National Cardiovascular Center Harapan Kita

Jl. Let. Jend. S. Parman, Kav 87, Slipi

West Jakarta, 11420, Indonesia

Work: (+62)21 5684085 ext. 2807

Mobile: (+62)813 8570 2548

E-mail: eva.marwali@pjnhk.go.id

Abstract word count : **300**

Total word count : **2497**

Conflict of interest : none for this paper

Confidential: For Review Only

Abstract

Background: The impact of the COVID-19 pandemic on pediatric populations varied between high (HICs) versus low to middle income countries (LMICs). We sought to investigate differences in pediatric clinical outcomes and identify factors contributing to disparity between countries.

Methods: The International Severe Acute Respiratory and Emerging Infections Consortium (ISARIC) COVID-19 database was queried to include children under 19 years of age admitted to hospital from January 2020 through April 2021 with suspected or confirmed COVID-19 diagnosis. Bivariate and multivariate analysis of contributing factors for mortality were assessed by country group (HICs vs. LMICs) as defined by the World Bank criteria.

Results: A total of 12,860 children (3,819 from 21 HICs and 9,041 from 15 LMICs) participated in this study. Of these, 8,961 were laboratory-confirmed and 3,899 suspected COVID-19 cases. About 52% of LMICs children were Black and more than 40% were infants and adolescent. Overall in-hospital mortality rate (95% Confidence Interval [CI]) was 3.3% [3.0 - 3.6%], higher in LMICs than HICs (4.0% [3.6 – 4.4%] and 1.7% [1.3 -2.1%], respectively). There were significant differences between country income groups in intervention profile, with higher use of antibiotics, antivirals, corticosteroids, prone positioning, high flow nasal cannula, non-invasive and invasive mechanical ventilation in HICs. Out of the 439 mechanically ventilated children, mortality occurred in 106 (24.1%) subjects, which was higher in LMICs than HICs (89 (43.6%) vs. 17 (7.2%) respectively). Pre-existing infectious comorbidities (tuberculosis and HIV) and some complications (bacterial pneumonia, acute respiratory distress syndrome, and myocarditis) were significantly higher in LMICs compared with HICs. On multivariable analysis, LMIC as country income group was associated with increased risk of mortality (adjusted HR 4.74 [3.16-7.10]).

Conclusion: Differences in clinical outcomes of pediatric COVID-19 cases between LMICs and HICs may be attributable to differences in patient demographics, complications, and access to supportive and treatment modalities.

Funding: National Institute for Health Research (NIHR), UK Medical Research Council, Wellcome Trust, Department for International Development, Bill & Melinda Gates Foundation, EU Platform for European Preparedness Against (Re-)emerging Epidemics, NIHR Health Protection Research Unit (HPRU) in Emerging and Zoonotic Infections at University of Liverpool, NIHR HPRU in Respiratory Infections at Imperial College London.

Keywords: child mortality, health care rationing, health resources, SARS-CoV-2

Confidential: For Review Only

Introduction

The clinical presentation, severity, and outcomes of acute COVID-19 are different in children compared to adults. While a higher proportion of children are asymptomatic or less severely ill than in many adult reports, severe manifestations do occur.[1] While cardiac compromise in the form of Multisystem Inflammatory Syndrome in Children (MIS-C) is often described, acute respiratory distress syndrome and other organ dysfunction also occurs in children.[2,3] The risk factors for severe disease in in pediatrics are incompletely understood.[4] Furthermore, there is a lack of global data to improve understanding of the COVID-19 burden in children who live in low to middle income countries (LMICs) versus those in high-income (HICs) sites.[5]

One systematic review that summarized the difference in pediatric COVID-19 morbidity and mortality in HICs and LMICs has been published.[6] However, most studies' samples analyzed fewer than 100 patients and over half the data came from the USA and China. Furthermore, due to heterogeneous reporting of data in the included studies, the authors were limited in their ability to pool or compare data on clinical features and outcomes. A recent scoping review reported that ethnicity and socioeconomic condition were underrepresented in COVID-19 epidemiological studies.[7] We aimed to investigate the differences in survival of pediatric patients in LMICs and HICs and the factors that may contribute to such differences between regions.

Methods

Data was collected from hospitalized patients under 19 years of age with confirmed or clinically suspected COVID-19 between January 1, 2020 and March 31, 2021 admitted to institutions across the globe contributing to the International Severe Acute Respiratory and Emerging Infections Consortium (ISARIC) database according to ISARIC/ WHO Clinical Characterization Protocol for Severe Emerging Infection.[1,8] Fields for analysis were extracted from the complete dataset gathered from Research Electronic Data Capture (REDCap, version 8.11.11, Vanderbilt University, Nashville, Tennessee).[8]

Variables of interest were classified into four domains: comorbidities, presenting signs and symptoms, complications, and treatments. Comorbidity was defined as any history of pre-existing medical conditions that were not otherwise related to COVID-19 natural history and reported at admission date. Complications referred to any medical condition detected during patients' stay that was not present at admission. Therapies included were drugs, oxygen, and use of other treatments such as invasive mechanical ventilation. Ethnicity was collapsed into five categories (Black or African American, White, Asian, Mixed/others and missing/ unknown) following the Centers for Disease Control

Confidential: For Review Only

and Prevention (CDC) National Health Interview Survey (NHIS) glossary.[9] Country income groups were dichotomized into HICs and LMICs according to the latest World Bank classification.[10]

Date of admission was defined as the date of hospitalization. Descriptive statistics were described as frequencies (n) and proportions for categorical data, mean ± standard deviation (SD) or median (interquartile range [IQR]) for continuous data, and number of available data (N) for each variable. Demographic characteristics, comorbidities, complications, and treatments were compared between country income groups using Chi-square or Fischer's Exact test as indicated. Kaplan-Meier survival curves were plotted and compared using the log-rank test. Multivariable Cox proportional hazards regression models were fitted to identify mortality predictors. In hospital survival analysis was performed to obtain 28- and 90-day survival rates. Intensive care unit (ICU) admission and invasive mechanical ventilation (IMV) requirement served as proxies for morbidity. Time-to-event analyses were performed to identify morbidity and mortality-defining periods: 1) from hospital admission until ICU admission, 2) ICU admission to first intubation, 3) ICU admission to ICU discharge or death, 4) ICU discharge to hospital discharge or death. A p-value of <0.05 was considered statistically significant. Statistical analyses were performed using SPSS 25 (IBM Corp, Armonk, NY).

Results

There were 12,860 children, originating from 36 countries, 15 of which (41.7%) were LMICs and 21 (58.3%) HICs. Seventy percent (n=9041) of participants were from LMICs and 29.7% (n=3819) cases were from HICs. The majority of participants were contributed from 670 cites in South Africa (59.3%) and 346 sites in the United Kingdom (UK) (24.1%), followed by Malaysia (4.9%), Poland (2,4%), and Malawi (2.3%) (**Figure 1**). COVID-19 status was laboratory-confirmed in 69.7%; this rate was higher in HICs (73.0%) in comparison to that in LMICs (68.3%). **(Table 1)**

Adolescents aged between 12 and 17 years, were the largest age group in LMICs (25.6%), and overall (24.0%), followed by infants younger than one year of age (21%). Infants were the largest group in HICs (29.4%). Males (50.8%) and females (49.2%) were similarly represented. Black or African American participants formed more than one-third of the study population and 52.% of LMICs children (**Table 1**)

In total, 425 (3.3%) participants died. The mortality rate was higher in in LMICs compared to HICs (1.7% vs.4%) (**Table 1**). The distribution of the admission and mortality rates across the study period was presented in **Figure 2** as 3 country groups UK, South Africa and other countries. The curves showed bimodal with peaks of mortality rates

Confidential: For Review Only

coinciding with admissions during July 2020 and January 2021. Available case data in early 2020 was limited compared to later time points. In contrast to South Africa and other countries, mortality rate in the UK did not rise significantly with the first and second wave of case admissions during April-May 2020 and October 2020-January 2021.

Children in LMICs had significantly greater and earlier mortality (aHR [95% CI] 4.73 [3.16-7.10], $p < 0.001$). The 28 and 90-days survival among all participants were 96.7% (10,339/10,692) and 96.5% (10,642/11,024), respectively. Survival was higher at both 28 (98.3% (3049/3101)) and 90 days (98.1% (3137/3199)) in HICs than LMICs (96.0% (7290/7591) and 95% (7505/7825), respectively).

The availability of information on comorbidities varied between countries of origin. The prevalence of several comorbidities was significantly higher in HICs including chronic neurological disease, seizures, diabetes and chronic cardiac disease. Infectious diseases such as tuberculosis and HIV/AIDS were significantly higher in LMICs. (**Table 2)**

The most common presenting symptoms overall include fever (20%), cough (16.1%), and shortness of breath(10.5%). Between-group's comparisons showed that cough (HIC 14.0% vs. LMIC 26.1%), shortness of breath (9.2% vs. 16,4%) , runny nose (4.2% vs. 8.6%), loss of smell or taste (0.7% vs. 2.9%), anorexia (0.1% vs. 0.8%), and inability to walk (0.1% vs. 0.2%) were more common among patients in LMICs.

Complications during hospitalization are shown in **Table 3**. Patients in LMICs had more bacterial and cryptogenic pneumonia and ARDS, as well as other organ involvement such as brain with stroke or heart with cardiac arrest. The rates of complications were generally higher than that of HICs, except for cardiac arrhythmia and disseminated intravascular coagulation in which HICs rates were statistically higher.

The two most commonly administered therapies were antibiotics in 41.2% of participants, followed by corticosteroids in 11.4%. Antivirus was administered in 5.3% of subjects, with higher percentage observed in HIC, especially of Remdesivir which is more than twice that of LMIC. Adjunctive and supportive treatments were generally performed more often in HICs. No participants in LMICs were treated with extracorporeal membrane oxygenation, as compared to 10 participants in HICs (**Table 4**).

Participants in LMICs were most often admitted to the ICU within the first day of admission, with those who died being admitted earlier than survivors (LMICs vs. HICs: 0 [0 – 1] vs. 0 [0 – 2.5] day respectively, $p = 0.03$) . While time to invasive mechanical ventilation (IMV) was not significantly different for survivors vs. non-survivors in either LMIC or HICs, most received IMV in the first day of admission and all within a maximum of 3.5 days. Time from

Confidential: For Review Only

ICU admission to ICU discharge or death was significantly shorter in survivors vs. non-survivors although non survivors in HICs had longer stays than in LMICs (3 [1 – 6] vs. 9.5 [4.5–19.2] days respectively in HIC and 0 [0 – 4] vs. 4 [0.7 – 7.2] days respectively in LMIC, $p < 0.05$), Mortality was significantly higher ($p < 0.001$) in LMICs compared to HICs for participants who received invasive mechanical ventilation (IMV) (43.6% vs. 7.3%) or who required ICU admission (16.7% vs. 3.5). Nearly 2.3% and 6.1% participants died without ICU and/or IMV support in LMICs in comparison to the respective 1.3% and 1.4% in HICs (**Table 5**).

Multivariate analysis was done to evaluate factors associated with mortality. Significant risk factors found were: aged <1 year (adjusted hazard ratio (aHR) [95% Confidence Interval (CI)] = 1.80 [1.01- 3.22]), low-middle income group (4.73 [3.16 - 7.10]), comorbidities such as chronic kidney disease (3.74 [2.20-6.35]) or cardiac disease (2.42 [1.50-3.91]), and invasive mechanical ventilator requirement (3.46 [2.27 - 5.28]) or exposure to antibiotics (2.07 [1.34 - 3.22]). The use of antiviral agents (aHR= 0.55 [0.32 - 0.96]) was the only factor inversely associated with mortality (**Figure 3**).

Discussion

We present a large international cohort of children hospitalized with COVID- 19. We found that mortality was significantly higher in LMICs in comparison to HICs. Disparate care patterns were also observed, with patients in LMICs reported to receive most adjunctive and supportive therapies less frequently than patients in HICs. While these findings may represent differences in practice, they may also represent variation in available supports for children based on income status of the country. Such disparities have been described in adult COVID-19 patients, but limited data exists for children. Prior reports have focused on specific aspects of illness such as infection or cardiac dysfunction, have included small cohorts of children, or are limited to certain countries or regions.[11-20]

While the findings may be criticized as mainly representing data from 2 countries, the UK and South Africa, these countries are good examples of HICs and LMICs. Further, as data supplied from the UK and South Africa comes from national COVID-19 research databases recruiting from a high number of sites in the UK and South Africa, and in this sense may be more representative of country income differences, as opposed to enrolment of single sites (e.g. a national referral centre) in different countries. The inclusion of children from many other countries, although relatively small cohorts form each country in comparison, does allow understanding of care patterns in areas around the world.

Confidential: For Review Only

More participants from HICs could be admitted to ICU and received IMV than LMICs patients. While the small numbers available for analysis in some categories limit our confidence in these findings, in LMICs they do given IMV and dying within shorter periods of time than HICs. Not only were children in LMICs hospitalized with COVID-19 more likely to die, they were also shown to die earlier in their hospitalization. Despite possible confounding effects from missing data relating to severity of illness at presentation (vital signs, organ failure scores), a positive association between LMICs and mortality were consistently observed in analysis of children admitted to the ICU and those receiving IMV.

Several independent risk factors for mortality were identified in addition to country economic group. Mortality was lowest for patients aged between 1 and 5 years and higher among patients of age <1 or >5 years. This finding confirmed the U-shaped mortality pattern shown in several other reports, although infancy is not always recognized as a risk factor in small studies.[1,21,22] Comorbidities such as chronic kidney and cardiac diseases were also shown to be independent risk factors as reported by others.[19,23,24] These risk factors were more prevalent in HICs and thus did not seem to be associated with the high mortality rates noted in LMICs but this may reflect under-diagnosis possibly due to lack of diagnostic resources in some LMICs.[20] Higher rates of certain comorbidities, many infectious in nature, is another possible cause of the relationship between LMICs and mortality. Chronic respiratory failure has been associated with death in COVID-19 adult patients, with some evidence that this is occurs in pediatric cases as well. Our data also provided information on tuberculosis, which has not specifically identified as comorbidity in children with COVID in other reports. Similarly, data on the impact of HIV in children is sparse and our review finds this to be an important risk factor and more prevalent in LMICs.[23,25]

More patients receiving antiviral therapy were found in HICs vs LMICS. In fact, remdesivir, which is recommended for severe hospitalized COVID, was used in exceptionally lower percentage of LMIC subjects. We can only speculate that period of this study occurred when evidence based on antiviral efficacy was still scarce especially in children, or it may indicate lack of access to drug or lack familiarity with recommendations.[26] The recommendations for antiviral use in children with severe COVID from the National Institutes of Health (NIH) have suggested use for patients over the age of 12 years; this recommendation would not have applied to infants who had a higher risk of mortality in our study.[27,28] Further investigation of the impact of antivirals in children of all ages should be considered. The efficacy and the cost benefit of these expensive medications in resource-limited sites are needed; if valuable, improving access should then be at the core of discussions.

The use of other therapies listed in Supplementary **Table 3** also highlight the differences between LMICs and HICs. There was less use of many adjunct therapies associated with outcomes in adult studies such as prone positioning,

https://mc.manuscriptcentral.com/bmjpo

Confidential: For Review Only

high flow nasal cannula, antibiotics, steroids in LMIC sites. Whether these differences were the result of lack of availability of therapies or other regional factors cannot be determined, but it seems likely that limitation to access may influence practice. The high rate of mortality in patients outside the ICU and who died without invasive mechanical ventilation suggests that limitations to ICU beds or ventilators in LMICs likely play a large role in the excess death rates reported as compared to HICs.[29,30]

The higher prevalence of complications of respiratory disease such as ARDS, bacterial and cryptogenic organizing pneumonia (COP), and the impact of organ dysfunction outside the lung such as increased rates of myocarditis, pericarditis, endocarditis, meningitis, encephalitis, stroke and cardiac arrest observed in LMICs are also likely factors in the high death rates. Patients with MIS-C were not specifically reported in the time period of this report.

Our study has the strength of a common reporting format in participating centers around the world. We describe a relatively large number of children and are able to provide both comparisons of patient characteristics and outcomes and evaluate risk factors for outcomes using common definitions. Limitation of this study includes a predominance of patients from one LMICs and one HICs -South Africa and UK, potentially limiting the generalizability of our findings to all countries. In addition, we did not adjusted for pandemic era. Inevitably we have missing data for a number of variables which limits the effective sample size of analyses examining relationships with patient characteristics and outcomes.

In conclusion, we found many differences in characteristics, treatments and outcomes among children from LMICs and HICs with infants had higher death rates than other children. Patients less frequently receive invasive mechanical ventilation and other supportive therapies in LMICs, which likely represents disparities in access to healthcare that influence outcomes. Reducing the gap in our ability to care for sick children in LMICs versus HICs will inevitably improve global outcomes during both pandemic and interpandemic periods.

Data sharing

Data and material access are available upon data access committee agreement fulfilling the full terms (https://www.iddo.org/document/covid-19-data-access-guidelines). Applications are welcome via the Independent Data and Material Access Committee (https://isaric4c.net).

Declaration of interests

Competing interests: All authors have completed the ICMJE uniform disclosure form at www.icmje.org/coi_disclosure.pdf and declare no competing interest.

Confidential: For Review Only

Author Contributions

The author contributions are as follows; study conception and design: EMM, AK, SY, DKW, MR, ICV, HJC, WJ, LB, MM, MGS, OVS, MKV, JP, SM, RF, AMG, AS, MDP, AR, CK, BPG, BC, LM, PLO, HD; coordination and collection of data: EMM, AK, SY, DKW, MR, ICV, HJC, WJ, LB, MM, MGS, OVS, MKV, JP, SM, RF, AMG, AS, MDP, AR, CK, BPG, BC, LM, PLO, HD; statistical analysis or interpretation of data: EMM, AK, SY, DKW, MR, ICV, HJC, WJ, LB, MM, MGS, OVS, MKV, JP, SM, RF, AMG, AS, MDP, AR, CK, BPG, BC, LM, PLO, HD; drafting the manuscript: EMM, MR, ICV; critical review and revision of the manuscript for important intellectual insight: EMM, AK, SY, DKW, MR, ICV, HJC, WJ, LB, MM, MGS, OVS, MKV, JP, SM, RF, AMG, AS, MDP, AR, CK, BPG, BC, LM, PLO, HD; study supervision: EMM, BC, LM. All authors drafted the manuscript from important intellectual viewpoints and approved the final version. Furthermore, all authors agreed to be accountable for all aspects of the work in ensuring that questions related to the accuracy or integrity of any part of the work are appropriately investigated and resolved. The corresponding author confirmed that all authors meet authorship criteria according to ICMJE.

Confidential: For Review Only

References

1. Docherty A B, Harrison E M, Green C A, Hardwick H E, Pius R, Norman L et al. Features of 20 133 UK patients in hospital with covid-19 using the ISARIC WHO Clinical Characterisation Protocol: prospective observational cohort study. BMJ 2020;369:m1985.
2. Han X, Li X, Xiao Y, Yang R, Wang Y, Wei X. Distinct Characteristics of COVID-19 Infection in Children. *Front Pediatr*. 2021;9:619738.
3. Borrelli M, Corcione A, Castellano F, Fiori Nastro F, Santamaria F. Coronavirus Disease 2019 in Children. *Front Pediatr*. 2021;9:668484.
4. Fialkowski A, Gernez Y, Arya P, Weinacht KG, Kinane TB, Yonker LM. Insight into the pediatric and adult dichotomy of COVID-19: Age-related differences in the immune response to SARS-CoV-2 infection. *Pediatr Pulmonol*. 2020 Oct;55(10):2556–2564.
5. Shadmi E, Chen Y, Dourado I, et al. Health equity and COVID-19: global perspectives. *Int J Equity Health*. 2020;19(1):104. doi:10.1186/s12939-020-01218-zKitano T, Kitano M, Krueger C, et al. The differential impact of pediatric COVID-19 between high-income countries and low- and middle-income countries: A systematic review of fatality and ICU admission in children worldwide. *PLoS ONE* 2021;16(1):e0246326.
6. Kitano T, Kitano M, Krueger C, et al. The differential impact of pediatric COVID-19 between high-income countries and low- and middle-income countries: A systematic review of fatality and ICU admission in children worldwide. *PLoS ONE* 2021;16(1):e0246326.
7. Bernardino FBS, Alencastro LC da S, Silva RA da, Ribeiro AD do N, Castilho GR de C, Gaíva MAM. Epidemiological profile of children and adolescents with COVID-19: a scoping review. *Rev bras enferm* 2021;74(Suppl 1):e20200624.
8. Abdukahil SA, Abe R, Abel L, et al. COVID-19 symptoms at hospital admission vary with age and sex: results from the ISARIC prospective multinational observational study. *Infection* 2021;49(5):889.
9. National Center for Health Statistics. National Health Interview Survey Glossary. Centers for Disease Control and Prevention.
10. The World Bank. The world by income and region. Accessed March 3, 2022. https://datatopics.worldbank.org/world-development-indicators/the-world-by-income-and-region.html.
11. Swann OV, Holden KA, Turtle A, et al. Clinical characteristics of children and young people admitted to hospital with covid-19 in United Kingdom: prospective multicentre observational cohort study. *BMJ* 2020;370:m3249.
12. Ghisolfi S, Almås I, Sandefur JC, et al. Predicted COVID-19 fatality rates based on age, sex, comorbidities and

Confidential: For Review Only

health system capacity. *BMJ Glob Health* 2020;5:e003094.

13. Ludvigsson JF. Systematic review of COVID-19 in children shows milder cases and a better prognosis than adults. *Acta Paediatr* 2020;00:1-8.
14. Chhibber-Goel J, Malhotra S, Krishnan NMA, Sharma A, et al. The profiles of first and second SARS-CoV-2 waves in the top ten COVID-19 affected countries. *J Glob Health* 2021;5:e2021082. doi:10.29392/001c.27143.
15. Delahoy MJ, Ujamaa D, Whitaker M, et al. Hospitalizations Associated with COVID-19 Among Children and Adolescents — COVID-NET, 14 States, March 1, 2020–August 14, 2021. *Morb Mortal Wkly Rep* 2021;70(36):1255–60.
16. Thelwall S, Aiano F, Harman K, et al. Risk of hospitalisation and death in children with SARS-CoV-2 delta (B.1.612.2) infection. *Lancet Child Adolesc Health* 2022. https://doi.org/10.1016/ S2352-4642(22)00096-7.
17. Liu X, Huang J, Li C, et al. The role of seasonality in the spread of COVID-19 pandemic. *Environ Res* 2021;195:110874.
18. Nachega JB, Sam-Agudu NA, Machekano RN, et al. Assessment of Clinical Outcomes Among Children and Adolescents Hospitalized With COVID-19 in 6 Sub- Saharan African Countries. *JAMA Pediatr* 2022 Mar; 176(3): e216436.
19. Gonzalez-Dambrauskas S, Vasquez-Hoyos P, Camporesi A, et al. Paediatric critical COVID-19 and mortality in a multinational prospective cohort. *The Lancet Regional Health Americas* 2022;12: 100272.
20. Oliveira EA, Colosimo EA, Silva ACS, et al. Clinical characteristics and risk factors for death among hospitalised children and adolescents with COVID-19 in Brazil: an analysis of a nationwide database. *Lancet Child Adolesc Health* 2021; 5: 559–68.
21. Khera N, Santesmasses D, Kerepesi C, et al. COVID-10 mortality rate in children is U-shaped. *Aging* 2021;13(16):19954–62.
22. O'Driscoll M, Dos Santos GB, Wang L, et al. Age-specific mortality and immunity patterns of SARS-CoV-2. *Nature* 2020. https://doi.org/10.1038/s41586-020-2918-0
23. Tsankov BK, Allaire JM, Irvine MA, et al. Severe COVID-19 Infection and Pediatric Comorbidities: A Systematic Review and Meta-Analysis. *Int J Infect Dis* 2021;103:246–56.
24. Marin BG, Aghagoli G, Lavine K, et al. Predictors of COVID-19 severity: A literature review. *Rev Med Virol* 2021;31(1):1–10.
25. Song W, Zhao J, Zhang Q, et al. COVID-19 and Tuberculosis Coinfection: An Overview of Case Reports/Case Series and Meta-Analysis. *Front Med* 2021;8:657006.

Confidential: For Review Only

26. Panda PK, Sharawat IK, Natarajan V, et al. COVID-19 treatment in children: A systematic review and meta-analysis. *J Family Med Prim Care* 2021;10:3292–302.

27. COVID-19 Treatment Guidelines Panel. Coronavirus Disease 2019 (COVID-19) Treatment Guidelines. National Institutes of Health. Available at https://www.covid19treatmentguidelines.nih.gov/. Accessed 1 Apr 2022.

28. La Tessa A, Motisi MA, Marseglia GL, et al. Use of remdesivir in children with COVID-19 infection: a quick narrative review. *Acta Biomed* 2021; 92(7): e2021524.

29. Austin S, Murthy S, Wunsch H, et al. Access to urban acute care services in high- vs. middle-income countries: an analysis of seven cities. *Intensive Care Med* 2014;40:342–352.

30. Murthy S, Leligdowicz A, Adhikari NKJ. Intensive Care Unit Capacity in Low-Income Countries: A Systematic Review. *PLoS ONE* 2015;10(1):e0116949.

Confidential: For Review Only

Table 1. Baseline characteristics of all study participants (n = 12,860)

		Country Income						p
		HICs (N=3819)		LMICs (N=9041)		Total		
		n	%	n	%	n	%	
Age*	<1 year	1124	29.4	1574	17.4	2698	21.0	<0.001
	1 to <5 years	707	18.5	1887	20.9	2594	20.2	
	5 to <12 years	796	20.8	1621	17.9	2417	18.8	
	12 to <17 years	770	20.2	2311	25.6	3081	24.0	
	≥17 years	422	11.1	1648	18.2	2070	16.1	
Sex*	Male	2026	53.1	4497	49.8	6523	50.8	0.002
	Female	1788	46.9	4537	50.2	6325	49.2	
Ethnicity *	Black or African American	223	5.8	4747	52.5	4970	38.6	<0.001
	Missing and unknown	1221	32.0	3509	38.8	4730	36.8	
	White	1624	42.5	186	2.1	1810	14.1	
	Asian	561	14.7	168	1.9	729	5.7	
	Mixed and others	190	5.0	431	4.8	621	4.8	
COVID-19 status*	Confirmed	2786	73	6175	68.3	8961	69.7	<0.001
	Suspect	1033	27	2866	31.7	3899	30.3	
Outcome*	Discharged	3423	89.6	7967	88.1	11390	88.6	<0.001
	Still in hospital	140	3.7	357	3.9	497	3.9	
	Transferred	192	5.0	356	3.9	548	4.3	
	Death	64	1.7	361	4.0	425	3.3	

Significant with p-value <0.05 using Chi-square test (*).

HICs: high-income countries, LMICs : low- to middle-income countries.

Confidential: For Review Only

Table 2. Comparison of comorbidities between country income groups

	Country Income									p
	HICs			LMICs			Total			
	N	n	%	N	n	%	N	n	%	
Chronic neurological disorder*	2588	191	7.4	902	14	1.6	3490	205	5.9	0.001
Seizure*	2490	111	4.5	899	16	1.8	3389	127	3.7	0.001
Smoking	2091	82	3.9	1509	46	3.0	3600	128	3.6	0.215
Diabetes*	2592	95	3.7	4468	149	3.3	7060	244	3.5	0.001
Chronic cardiac disease*	2587	141	5.5	3578	48	1.3	6165	189	3.1	0.001
Obesity*	2438	90	3.7	1736	39	2.2	4174	129	3.1	0.001
Tuberculosis*	290	-	-	3649	102	2.8	3939	102	2.6	0.001
Chronic hematological disease*	2475	77	3.1	899	9	1.0	3374	86	2.5	0.001
HIV/AIDS*	2562	1	<0.01	4382	142	3.2	6944	143	2.1	0.001
Rare diseases and inborn errors of metabolism	1823	36	2.0	0	-	-	1823	36	2.0%	0.715
Malnutrition	2539	35	1.4	901	26	2.9%	3440	61	1.8%	0.412
Malignant neoplasm*	2571	64	2.5	4164	27	0.6%	6735	91	1.4%	0.001
Rheumatological disorder*	2491	45	1.8	898	2	0.2%	3389	47	1.4%	0.001
Chronic kidney disease*	2596	59	2.3	4147	28	0.7	6743	87	1.3	0.001
Liver disease	2623	12	0.5	902	-	-	3525	12	0.3	0.866

Significant with p-value <0.05 using Chi-square test (*).

HICs: high-income countries; LMICs : low- to middle-income countries; N= denominators; n= total patients with comorbidities.

Confidential: For Review Only

15

Table 3. Comparison of complications between country income groups

	Country Income									
	HICs			LMICs			Total			p
	N	n	%	N	n	%	N	n	%	
Bacterial pneumonia*	3497	115	3.3	1107	102	9.2	4604	217	4.7	<0.001
ARDS*	3153	31	1.0	4972	267	5.4	8125	298	3.7	<0.001
AKI	3624	110	3.0	1410	49	3.5	5034	159	3.2	0.477
Seizure	3627	103	2.8	1412	39	2.8	5039	142	2.8	0.958
Pleural effusion	3511	82	2.3	1110	36	3.2	4621	118	2.6	0.118
Myocarditis and pericarditis*	2858	42	1.5	427	22	5.2	3285	64	1.9	<0.001
Bronchiolitis	3620	57	1.6	1412	21	1.5	5032	78	1.6	0.922
Cardiac arrhythmia*	3635	70	1.9	1407	13	0.9	5042	83	1.6	0.017
Endocarditis*	692	5	0.7	487	14	2.9	1179	19	1.6	0.008
Cardiac arrest*	3535	19	0.5	1412	55	3.9	4947	74	1.5	<0.001
DIC*	3498	48	1.3	4668	58	1.2	8166	104	1.3	0.850
Meningitis and encephalitis*	3621	16	0.4	1413	20	1.4	5034	36	0.7	<0.001
Pneumothorax	3530	15	0.4	1109	9	0.8	4639	24	0.5	0.185
Stroke/cerebrovascular complication[a]	3527	7	0.2	1109	11	1.0	4636	18	0.4	0.001
Pulmonary Embolism	1928	4	0.2	293	2	0.7	2221	6	0.3	0.182
Cardiac ischemia	3510	7	0.2	1110	4	0.4	4620	11	0.2	0.309
Myocardial infarction	312	1	0.3	298	-	-	610	1	0.2	1.000
Sepsis	-	-	-	3862	8	0.2	3862	8	0.2	-
DVT	1606	1	0.1	3	-	-	1609	1	0.1	1.000
COP[a]	3398	-	-	1110	5	0.5	4508	5	0.1	0.001

Significant with p-value <0.05 using Chi-square test (*) or Fischer's Exact test ([a])
AKI: acute kidney injury; ARDS: acute respiratory distress syndrome; COP: cryptogenic organizing pneumonia; DIC: disseminated intravascular coagulation; DVT: deep vein thrombosis; HICs: high-income countries; LMICs : low- to middle-income countries; N= denominators; n= total patients with complications.

Confidential: For Review Only

16

Table 4. Treatment profile of study participants.

	Country Income									p
	HICs			LMICs			Total			
	N	n	%	N	n	%	N	n	%	
Antibiotics*	3685	2189	59.4	4349	1122	25.8	8034	3311	41.2	<0.001
Corticosteroid*	3663	564	15.4	4905	417	8.5	8568	981	11.4	<0.001
HFNC*	3380	282	8.3	3967	147	3.7	7347	429	5.8	<0.001
IMV*	3620	235	6.5	4684	204	4.3	8304	439	5.3	<0.001
Antivirus*	3672	271	7.4	4341	172	4.0	8013	443	5.3	<0.001
Remdesivir	3672	96	2.6	4341	17	0.4	8013	113	1.4	
Neuraminidase inhibitor	3672	32	0.9	4341	23	0.5	8013	113	55	
Inotropic/vasopressor*	3451	201	5.8	4961	174	3.5	8412	375	4.4	<0.001
Prone positioning*	3532	64	1.8	4647	39	0.8	8179	103	1.2	<0.001
Anticoagulant	3819	50	1.3	9041	88	1.0	12860	138	1.1	0.091
NIV*	3627	53	1.5	4966	17	0.3	8593	70	0.8	<0.001
RRT	3573	19	0.5	4334	27	0.6	7907	46	0.6	0.084
ECMOa	3608	10	0.3	1086	-	-	4694	10	0.2	<0.001

Significant with p-value <0.05 using Chi-square test (*) or Fischer's Exact test ([a])
ECMO: extracorporeal membrane oxygenation; HFNC: high-flow nasal cannula; IMV: invasive mechanical ventilation; NIV: non-invasive ventilation; RRT: renal replacement therapy; HICs: high-income countries; LMICs : low- to middle-income countries; N= denominators; n= total patients with treatments.

17

Only

Table 5. Mortality based on ICU admission and invasive mechanical ventilation use.

		Mortality based on Country Income								
		HICs			LMICs			Total		
		N	n	%	N	n	%	N	n	%
ICU*	Yes	679	24	3.5	1037	173	16.7	1716	197	11.5
	No	3140	40	1.3	8004	188	2.3	11144	228	2.0
IMV*	Yes	235	17	7.2	204	89	43.6	439	106	24.1
	No	3385	47	1.4	4480	272	6.1	7865	319	4.0

Significant with p-value <0.05 using Chi-square test (*)
HICs: high-income countries; LMICs: low- to middle-income countries; ICU: intensive care unit; IMV: invasive mechanical ventilation; N= denominator (total patients with or without ICU/ IMV); n= total mortality.

Confidential: For Review Only

18

ISARIC Clinical Characterisation Group

Sheryl Ann Abdukahil, Nurul Najmee Abdulkadir, Laurent Abel, Lara Absil, Subhash Acharya, Diana Adrião, Younes Ait Tamlihat, Ernita Akmal, Eman Al Qasim, Tala Al-dabbous, Beatrice Alex, Kévin Alexandre, Huda Alfoudri, Kazali Enagnon Alidjnou, Clotilde Allavena, Nathalie Allou, Rita Alves, Joana Alves Cabrita, Maria Amaral, Nur Amira, Phoebe Ampaw, Claire Andréjak, Andrea Angheben, François Angoulvant, Séverine Ansart, Sivanesen Anthonidass, Carlos Alexandre Antunes de Brito, Ardiyan Apriyana, Yaseen Arabi, Carolline Araujo, Patrick Archambault, Jean-Benoît Arlet, Christel Arnold-Day, Elise Artaud-Macari, Diptesh Aryal, Muhammad Ashraf, Jean Baptiste Assie, Amirul Asyraf, Anika Atique, AM Udara Lakshan Attanyake, Johann Auchabie, Hugues Aumaitre, Adrien Auvet, Laurène Azemar, Cecile Azoulay, Benjamin Bach, Delphine Bachelet, Claudine Badr, J. Kenneth Baillie, Nazreen Abu Bakar, Mohanaprasanth Balakrishnan, Valeria Balan, Firouzé Bani-Sadr, Renata Barbalho, Wendy S. Barclay, Saef Umar Barnett, Audrey Barrelet, Marie Bartoli, Joaquín Baruch, Romain Basmaci, Muhammad Fadhli Hassin Basri, Jules Bauer, Diego Fernando Bautista Rincon, Abigail Beane, Alexandra Bedossa, Ker Hong Bee, Husna Begum, Sylvie Behilill, Albertus Beishuizen, Anna Beltrame, Marine Beluze, Nicolas Benech, Lionel Eric Benjiman, Dehbia Benkerrou, Delphine Bergeaud, José Luis Bernal Sobrino, Giulia Bertoli, Simon Bessis, Sybille Bevilcaqua, Karine Bezulier, Nowneet Kumar Bhat, Krishna Bhavsar, Farah Nadiah Bidin, Felwa Bin Humaid, Mohd Nazlin Bin Kamarudin, François Bissuel, Laurent Bitker, Jonathan Bitton, Mathieu Blot, Lucille Blumberg, Laetitia Bodenes, Debby Bogaert, Anne-Hélène Boivin, Isabela Bolaños, Pierre-Adrien Bolze, François Bompart, Diogo Borges, Raphaël Borie, Hans Martin Bosse, Elisabeth Botelho-Nevers, Lila Bouadma, Olivier Bouchaud, Sabelline Bouchez, Dounia Bouhmani, Damien Bouhour, Kévin Bouiller, Laurence Bouillet, Camile Bouisse, Anne-Sophie Boureau, Maude Bouscambert, Aurore Bousquet, Jason Bouziotis, Bianca Boxma, Marielle Boyer-Besseyre, Fernando Augusto Bozza, Axelle Braconnier, Cynthia Braga, Timo Brandenburger, Kathy Brickell, Marjolein Brusse-Keizer, Polina Bugaeva, Marielle Buisson, Erlina Burhan, Aidan Burrell, Ingrid G. Bustos, Denis Butnaru, André Cabie, Eder Caceres, Cyril Cadoz, Rui Caetano Garcês, Jose Andres Calvache, Valentine Campana, Paul Campbell, Pauline Caraux-Paz, Chiara Simona Cardellino, Sofia Cardoso, Filipe Cardoso, Filipa Cardoso, Nicolas Carlier, Thierry Carmoi, Marie-Christine Carret, François Martin Carrier, Gail Carson, Maire-Laure Casanova, Mariana Cascão, José Casimiro, Nidyanara Castanheira, Guylaine Castor-Alexandre, François-Xavier Catherine, Paolo Cattaneo, Roberta Cavalin, Minerva Cervantes-Gonzalez, Anissa Chair, Catherine Chakveatze, Adrienne Chan, Meera Chand, Christelle Chantalat Auger, Jean-Marc Chapplain, Julie Chas, Anjellica Chen, Matthew Pellan Cheng, Antoine Cheret, Thibault Chiarabini, Julian Chica, Suresh Kumar Chidambaram, Leong Chin Tho, Catherine Chirouze, Bernard Cholley, Marie-Charlotte Chopin, Ting Soo Chow, Hiu Jian Chua, Jonathan Chua, Barbara Wanjiru Citarella, Emma Clarke, Sara Clohisey, Alexandra Coelho, Megan Coles, Gwenhaël Colin, Pamela Combs, Marie Connor, Anne Conrad, Graham S. Cooke, Mary Copland, Hugues Cordel, Amanda Corley, Sabine Cornelis, Alexander Daniel Cornet, Arianne Joy Corpuz, Grégory Corvaisier, Emma Costigan, Camille Couffignal, Sandrine Couffin-Cadiergues, Roxane Courtois, Stéphanie Cousse, Rachel Cregan, Sabine Croonen, Claudina Cruz, Juan Luis Cruz Bermúdez, Jaime Cruz Rojo, Elodie Curlier, Paula Custodio, Ana da Silva Filipe, Charlene Da Silveira, Andrew Dagens, Heidi Dalton, Jo Dalton, Juliana Damas, Nick Daneman, Corinne Daniel, Emmanuelle A Dankwa, Jorge Dantas, Etienne De Montmollin, Rafael Freitas de Oliveira França, Thushan de Silva, Peter de Vries, Jillian Deacon, Alexa Debard, Marie-Pierre Debray, Nathalie DeCastro, William Dechert, Lauren Deconninck, Romain Decours, Eve Defous, Isabelle Delacroix, Eric Delaveuve, Karen Delavigne, Christelle Delmas, Pierre Delobel, Corine Delsing, Elisa Demonchy, Emmanuelle Denis, Dominique Deplanque, Pieter Depuydt, Diane Descamps, Mathilde Desvallées, Santi Dewayanti, Pathik Dhanger, Alpha Diallo, Sylvain Diamantis, André Dias, Rodrigo Diaz, Kévin Didier, Jean-Luc Diehl, Wim Dieperink, Jérôme Dimet, Vincent Dinot, Fara Diop, Alphonsine Diouf, Yael Dishon, Félix Djossou, Annemarie B. Docherty, Arjen M Dondorp, Maria Donnelly, Christl A. Donnelly, Chloe Donohue, Céline Dorival, Eric D'Ortenzio, James Joshua Douglas, Nathalie Dournon, Tom Drake, Murray Dryden, Murray Dryden, Claudio Duarte Fonseca, Vincent Dubee, François Dubos, Alexandre Ducancelle, Paul Dunand, Jake Dunning, Bertrand Dussol, Xavier Duval, Sim Choon Ean, Mohammed El Sanharawi, Brigitte Elharrar, Philippine Eloy, Isabelle Enderle, Chan Chee Eng, Ilka Engelmann, Vincent Enouf, Olivier Epaulard, Martina Escher, Hélène Esperou, Catarina Espírito Santo, Marina Esposito-Farese, João Estevão, Manuel

Confidential: For Review Only

Etienne, Nadia Ettalhaoui, Mirjam Evers, Marc Fabre, Isabelle Fabre, Amna Faheem, Arabella Fahy, Cameron J. Fairfield, Pedro Faria, Ahmed Farooq, Hanan Fateena, Karine Faure, Raphaël Favory, Jorge Fernandes, Marília Andreia Fernandes, François-Xavier Ferrand, Eglantine Ferrand Devouge, Joana Ferrão, Mário Ferraz, Bernardo Ferreira, Nicolas Ferriere, Céline Ficko, Claudia Figueiredo-Mello, Thomas Flament, Clara Flateau, Tom Fletcher, Victor Fomin, Tatiana Fonseca, Patricia Fontela, Simon Forsyth, Erwan Fourn, Robert A. Fowler, John F Fraser, Christophe Fraser, Marcela Vieira Freire, Ana Freitas Ribeiro, Stéphanie Fry, Valérie Gaborieau, Rostane Gaci, Jean-Charles Gagnard, Amandine Gagneux-Brunon, Carrol Gamble, Yasmin Gani, Noelia García Barrio, Esteban Garcia-Gallo, Denis Garot, Valérie Garrait, Nathalie Gault, Alexandre Gaymard, Eva Geraud, Louis Gerbaud Morlaes, Nuno Germano, Jade Ghosn, Carlo Giaquinto, Jess Gibson, Tristan Gigante, Morgane Gilg, Guillermo Giordano, Michelle Girvan, Valérie Gissot, Daniel Glikman, Petr Glybochko, Geraldine Goco, François Goehringer, Siri Goepel, Jean-Christophe Goffard, Jin Yi Goh, Marie Gominet, Bronner P. Gonçalves, Patricia Gordon, Isabelle Gorenne, Laure Goubert, Cécile Goujard, Tiphaine Goulenok, Pascal Granier, Christopher A. Green, Courtney Greene, William Greenhalf, Segolène Greffe, Fiona Griffiths, Albert Groenendijk, Anja Grosse Lordemann, Heidi Gruner, Yusing Gu, Jérémie Guedj, Martin Guego, Dewi Guellec, Anne-Marie Guerguerian, Daniela Guerreiro, Romain Guery, Anne Guillaumot, Laurent Guilleminault, Thomas Guimard, Marieke Haalboom, Daniel Haber, Hannah Habraken, Ali Hachemi, Nadir Hadri, Fakhir Haidri, Matthew Hall, Sophie Halpin, Ansley Hamer, Rebecca Hamidfar, Lim Yuen Han, Rashan Haniffa, Kok Wei Hao, Hayley Hardwick, Ewen M. Harrison, Janet Harrison, Lars Heggelund, Ross Hendry, Maxime Hentzien, Diana Hernandez, Astarini Hidayah, Rupert Higgins, Samuel Hinton, Hikombo Hitoto, Antonia Ho, Yi Bin Ho, Alexandre Hoctin, Isabelle Hoffmann, Wei Han Hoh, Peter Horby, Ikram Houas, Catherine L. Hough, Jimmy Ming-Yang Hsu, Jean-Sébastien Hulot, Samreen Ijaz, Arfan Ikram, Hajnal-Gabriela Illes, Patrick Imbert, Hugo Inácio, Yun Sii Ing, Elias Iosifidis, Sarah Isgett, Tiago Isidoro, Nadiah Ismail, Margaux Isnard, Danielle Jaafar, Salma Jaafoura, Julien Jabot, Clare Jackson, Nina Jamieson, Pierre Jaquet, Waasila Jassat, Coline Jaud-Fischer, Stéphane Jaureguiberry, Florence Jego, Anilawati Mat Jelani, Ong Yiaw Joe, Mark Joseph, Cédric Joseph, Swosti Joshi, Mercé Jourdain, Philippe Jouvet, Dafsah Juzar, Ouifiya Kafif, Florentia Kaguelidou, Neerusha Kaisbain, Thavamany Kaleesvran, Sabina Kali, Muhammad Aisar Ayadi Kamaluddin, Zul Amali Che Kamaruddin, Nadiah Kamarudin, Kong Yeow Kang, Pratap Karpayah, Todd Karsies, Christiana Kartsonaki, Anant Kataria, Kevin Katz, Seán Keating, Yvelynne Kelly, Sadie Kelly, Kalynn Kennon, Younes Kerroumi, Sharma Keshav, Antoine Khalil, Coralie Khan, Irfan Khan, Quratul Ain Khan, Sushil Khanal, Abid Khatak, Saye Khoo, Ryan Khoo, Denisa Khoo, Khor How Kiat, Peter Kiiza, Antoine Kimmoun, Detlef Kindgen-Milles, Paul Klenerman, Rob Klont, Gry Kloumann Bekken, Stephen R Knight, Robin Kobbe, Chamira Kodippily, Malte Kohns Vasconcelos, Arsène Kpangon, Vinothini Krishnan, Ganesh Kumar, Bharath Kumar Tirupakuzhi Vijayaraghavan, Pavan Kumar Vecham, Vinod Kumar, Neurinda Permata Kusumastuti, Demetrios Kutsogiannis, Marie Lachatre, Marie Lacoste, Marie Lagrange, Fabrice Laine, Olivier Lairez, Antonio Lalueza, Marc Lambert, Marie Langelot-Richard, Vincent Langlois, Marina Lanza, Cédric Laouénan, Samira Laribi, Delphine Lariviere, Stéphane Lasry, Odile Launay, Didier Laureillard, Yoan Lavie-Badie, Andy Law, Teresa Lawrence, Minh Le, Clément Le Bihan, Cyril Le Bris, Georges Le Falher, Lucie Le Fevre, Quentin Le Hingrat, Marion Le Maréchal, Soizic Le Mestre, Gwenaël Le Moal, Vincent Le Moing, Hervé Le Nagard, Paul Le Turnier, Ema Leal, Marta Leal Santos, Todd C. Lee, James Lee, Jennifer Lee, Heng Gee Lee, Biing Horng Lee, Yi Lin Lee, Gary Leeming, Laurent Lefebvre, Bénédicte Lefebvre, Benjamin Lefèvre, Sylvie LeGac, Jean-Daniel Lelievre, François Lellouche, Adrien Lemaignen, Véronique Lemee, Anthony Lemeur, Ha Sha Lene, Jenny Lennon, Marc Leone, Quentin Lepiller, François-Xavier Lescure, Olivier Lesens, Mathieu Lesouhaitier, Yves Levy, Bruno Levy, Claire Levy-Marchal, Erwan L'Her, Gianluigi Li Bassi, Geoffrey Liegeon, Wei Shen Lim, Kah Chuan Lim, Bruno Lina, Lim Lina, Guillaume Lingas, Sylvie Lion-Daolio, Samantha Lissauer, Marine Livrozet, Patricia Lizotte, Navy Lolong, Leong Chee Loon, Diogo Lopes, Anthony L. Loschner, Paul Loubet, Bouchra Loufti, Guillame Louis, Lee Lee Low, Marije Lowik, Jia Shyi Loy, Jean Christophe Lucet, Carlos Lumbreras Bermejo, Liem Luong, Dominique Luton, Ruth Lyons, Oryane Mabiala, Sara Machado, Moïse Machado, Gabriel Macheda, Hashmi Madiha, Guillermo Maestro de la Calle, Rafael Mahieu, Sophie Mahy, Mylène Maillet, Thomas Maitre, Nadia Malik, Fernando Maltez, Denis Malvy, Victoria Manda, Jose M. Mandei, Laurent Mandelbrot, Julie Mankikian, Edmund Manning, Aldric Manuel, Ceila Maria Sant`Ana Malaque, Daniel Marino, Samuel Markowicz, Laura Marsh, John Marshall, Celina Turchi Martelli, Guillaume Martin-Blondel, Martin Martinot, Ana Martins, Caroline Martins Rego, Eva Miranda Marwali, Marsilla

Confidential: For Review Only

Marzukie, David Maslove, Sabina Mason, Sobia Masood, Basri Mat Nor, Moshe Matan, Daniel Mathieu, Mathieu Mattei, Romans Matulevics, Laurence Maulin, Thierry Mazzoni, Colin McArthur, Anne McCarthy, Sarah E. McDonald, Kenneth A. McLean, Paul McNally, Cécile Mear-Passard, Ogechukwu Menkiti, Kusum Menon, France Mentré, Alexander J. Mentzer, Noémie Mercier, Emmanuelle Mercier, Antoine Merckx, Mayka Mergeay-Fabre, Laura Merson, António Mesquita, Agnès Meybeck, Alison M. Meynert, Vanina Meysonnier, Amina Meziane, Mehdi Mezidi, Céline Michelanglei, Isabelle Michelet, Nor Arisah Misnan, Tahira Jamal Mohamed, Nik Nur Eliza Mohamed, Asma Moin, Agostinho Monteiro, Claudia Montes, Shona C. Moore, Sarah Moore, Lina Morales Cely, Lucia Moro, Ben Morton, Hugo Mouquet, Clara Mouton Perrot, Julien Moyet, Caroline Mudara, Ng Yong Muh, Dzawani Muhamad, Jimmy Mullaert, Karl Erik Müller, Daniel Munblit, Marlène Murris, Srinivas Murthy, Himed Musaab, Himasha Muvindi, Gugapriyaa Muyandy, Nadège Neant, Nikita Nekliudov, Raul Neto, Anthony Nghi, Duc Nguyen, Alistair Nichol, Nurul Amani Mohd Noordin, Marion Noret, Nurul Faten Izzati Norharizam, Lisa Norman, Mahdad Noursadeghi, Karolina Nowicka, Saad Nseir, Nurnaningsih Nurnaningsih, Elsa Nyamankolly, Annmarie O'Callaghan, Katie O'Hearn, Agnieszka Oldakowska, Piero L. Olliaro, David S.Y. Ong, Jee Yan Ong, Wilna Oosthuyzen, Peter Openshaw, Saijad Orakzai, Claudia Milena Orozco-Chamorro, Linda O'Shea, Siti Zubaidah Othman, Nadia Ouamara, Rachida Ouissa, Eric Oziol, Maïder Pagadoy, Justine Pages, Massimo Palmarini, Prasan Kumar Panda, Lai Hui Pang, Nathalie Pansu, Aurélie Papadopoulos, Rachael Parke, Melissa Parker, Jérémie Pasquier, Bruno Pastene, Mohan Dass Pathmanathan, Juliette Patrier, Mical Paul, Christelle Paul, Jorge Paulos, William A. Paxton, Jean-François Payen, Kalaiarasu Peariasamy, Miguel Pedrera Jiménez, Florent Peelman, Nathan Peiffer-Smadja, Vincent Peigne, Ithan D. Peltan, Rui Pereira, Daniel Perez, Thomas Perpoint, Vincent Pestre, Michele Petrovic, Ventzislava Petrov-Sanchez, Gilles Peytavin, Scott Pharand, Walter Picard, Olivier Picone, Carola Pierobon, Djura Piersma, Carlos Pimentel, Raquel Pinto, Catarina Pires, Isabelle Pironneau, Lionel Piroth, Riinu Pius, Laurent Plantier, Hon Shen Png, Julien Poissy, Ryadh Pokeerbux, Maria Pokorska-Spiewak, Georgios Pollakis, Diane Ponscarme, Jolanta Popielska, Douwe F. Postma, Diana Póvoas, Sébastien Preau, Jean-Charles Preiser, Mark G. Pritchard, Gamage Dona Dilanthi Priyadarshani, Oriane Puéchal, Vilmaris Quinones-Cardona, Víctor Quirós González, Mohammed Quraishi, Christian Rabaud, Marie Rafiq, Rozanah Abd Rahman, Ahmad Kashfi Haji Ab Rahman, Giri Shan Rajahram, Nagarajan Ramakrishnan, José Ramalho, Ahmad Afiq Ramli, Blandine Rammaert, Grazielle Viana Ramos, Christophe Rapp, Aasiyah Rashan, Thalha Rashan, Menaldi Rasmin, Cornelius Rau, Tharmini Ravi, Stanislas Rebaudet, Sarah Redl, Brenda Reeve, Attaur Rehman, Jonathan Remppis, Martine Remy, Hongru Ren, Hanna Renk, Anne-Sophie Resseguier, Matthieu Revest, Oleksa Rewa, Luis Felipe Reyes, David Richardson, Laurent Richier, Siti Nurul Atikah Ahmad Ridzuan, Ana L Rios, Asgar Rishu, Patrick Rispal, Karine Risso, Stephanie Roberts, David L. Robertson, Olivier Robineau, Paola Rodari, Pierre-Marie Roger, Emmanuel Roilides, Amanda Rojek, Juliette Romaru, Mélanie Roriz, Manuel Rosa-Calatrava, Michael Rose, Andrea Rossanese, Bénédicte Rossignol, Patrick Rossignol, Stella Rousset, Carine Roy, Benoît Roze, Desy Rusmawatiningtyas, Clark D. Russell, Musharaf Sadat, Valla Sahraei, Maximilien Saint-Gilles, Stéphane Sallaberry, Charlotte Salmon Gandonniere, Hélène Salvator, Olivier Sanchez, Vanessa Sancho-Shimizu, Zulfiqar Sandhu, Pierre-François Sandrine, Oana Sandulescu, Shirley Sarfo-Mensah, Benjamine Sarton, Egle Saviciute, Parthena Savvidou, Yen Tsen Saw, Arnaud Scherpereel, Marion Schneider, Janet T. Scott, James Scott-Brown, Nicholas Sedillot, Jaganathan Selvanayagam, Mageswari Selvarajoo, Caroline Semaille, Malcolm G. Semple, Rasidah Bt Senian, Eric Senneville, Tânia Sequeira, Ary Serpa Neto, Pablo Serrano Balazote, Ellen Shadowitz, Syamin Asyraf Shahidan, Mohammad Shamsah, Shaikh Sharjeel, Catherine A. Shaw, Victoria Shaw, Ashraf Sheharyar, Mohiuddin Shiekh, Sally Shrapnel, Nassima Si Mohammed, Ng Yong Siang, Jeanne Sibiude, Louise Sigfrid, Benedict Sim Lim Heng, Karisha Sivam, Sue Smith, Morgane Snacken, Tze Vee Soh, Joshua Solomon, Tom Solomon, Agnès Sommet, Rima Song, Tae Song, Azlan Mat Soom, Albert Sotto, Edouard Soum, B. P. Sanka Ruwan Sri Darshana, Shiranee Sriskandan, Sarah Stabler, Ymkje Stienstra, Adrian Streinu-Cercel, Anca Streinu-Cercel, David Stuart, Jacky Y. Suen, Charlotte Summers, Deepashankari Suppiah, Andrey Svistunov, Sarah Syahrin, Jaques Sztajnbok, Shirin Tabrizi, Fabio S. Taccone, Lysa Tagherset, Shahdattul Mawarni Taib, Ewa Talarek, Kim Keat Tan, Yan Chyi Tan, Coralie Tardivon, Pierre Tattevin, M Azhari Taufik, Richard S. Tedder, Tze Yuan Tee, João Teixeira, Marie-Capucine Tellier, Sze Kye Teoh, François Téoulé, Olivier Terrier, Nicolas Terzi, Hubert Tessier-Grenier, Alif Adlan Mohd Thabit, Zhang Duan Tham, Suvintheran Thangavelu, Vincent Thibault, Simon-Djamel Thiberville, Benoît Thill, Jananee Thirumanickam, David Thomson, Emma C. Thomson, Surain Raaj Thanga

Confidential: For Review Only

Thurai, Ryan S. Thwaites, Paul Tierney, Peter S Timashev, Jean-François Timsit, Noémie Tissot, Jordan Zhien Yang Toh, Sia Loong Tonnii, Marta Torre, Margarida Torres, Tony Trapani, Théo Treoux, Cécile Tromeur, Tiffany Trouillon, Jeanne Truong, Christelle Tual, Sarah Tubiana, Jean-Marie Turmel, Lance C.W. Turtle, PG Ishara Udayanga, Andrew Udy, Timothy M. Uyeki, Luís Val-Flores, Amélie Valran, Marcel van den Berge, Job van der Palen, Paul van der Valk, Peter Van der Voort, Sylvie Van Der Werf, Jarne Van Hattem, Carolien van Netten, Ilonka van Veen, Noémie Vanel, Shoban Raj Vasudayan, Charline Vauchy, Shaminee Veeran, Aurélie Veislinger, Annelies Verbon, James Vickers, José Ernesto Vidal, César Vieira, Benoit Visseaux, Harald Vonkeman, Fanny Vuotto, Noor Hidayu Wahab, Suhaila Abdul Wahab, Nadirah Abdul Wahid, Steve Webb, Jia Wei, Katharina Weil, Tan Pei Wen, Sanne Wesselius, Murray Wham, Nicole White, Paul Henri Wicky, Aurélie Wiedemann, Evert-Jan Wils, Calvin Wong, Xin Ci Wong, Yew Sing Wong, Teck Fung Wong, Gan Ee Xian, Lim Saio Xian, Kuan Pei Xuan, Siti Rohani Binti Mohd Yakop, Yazdan Yazdanpanah, Nicholas Yee Liang Hing, Cécile Yelnik, Chian Hui Yeoh, Stephanie Yerkovich, Hodane Yonis, Obada Yousif, Saptadi Yuliarto, Marion Zabbe, Masliza Zahid, Maram Zahran, Nor Zaila Binti Zaidan, Maria Zambon, Konrad Zawadka, Nurul Zaynah, Hiba Zayyad, David Zucman, Mazankowski Heart Institute.

Confidential: For Review Only

Confidential: For Review Only

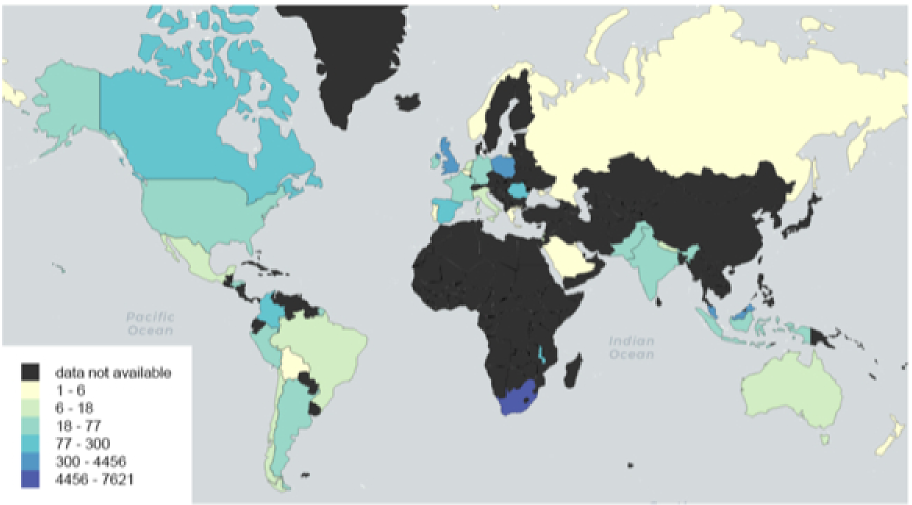

Figure 1. **Distribution of participants by country of origin**

Distribution of participants by country of origin.

108x65mm (150 x 150 DPI)

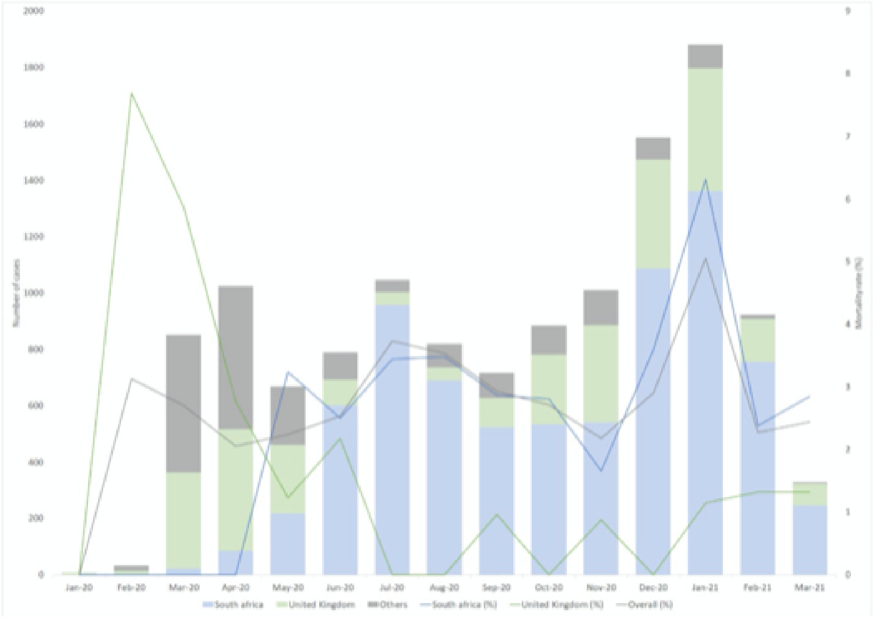

Figure 2. Total COVID-19 participants (bar) and mortality (line) rate trend throughout data collection period.

Total COVID-19 participants (bar) and mortality (line) rate trend throughout data collection period.

101x81mm (150 x 150 DPI)

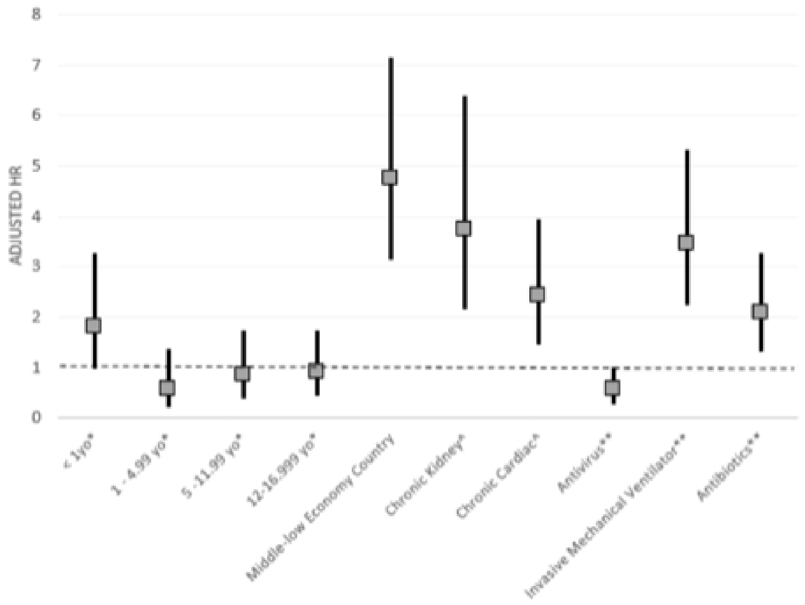

Figure 3. Adjusted Hazard Ratio and 95% Confidence Interval of mortality risk factors in all participants.

yo = years old; HR= Hazard Ratio

*) using age category ≥17 years as reference; ^) morbidity factors; **) COVID intervention factors

Adjusted Hazard Ratio and 95% Confidence Interval of mortality risk factors in all participants.
yo = years old; HR = Hazard Ratio
*Using age category >17 years as reference; ^) morbidity factors' **) COVID intervention factors.

96x81mm (150 x 150 DPI)

BMJ Paediatrics Open

PEDIATRIC COVID-19 MORTALITY: A DATABASE ANALYSIS OF THE IMPACT OF HEALTH RESOURCE DISPARITY

Journal:	*BMJ Paediatrics Open*
Manuscript ID	bmjpo-2022-001657.R1
Article Type:	Original research
Date Submitted by the Author:	03-Oct-2022
Complete List of Authors:	Marwali, Eva; National Cardiovascular Center, Pediatric Cardiac Intensive Care, National Cardiovascular Center Harapan Kita<br>Kekalih, Aria; Universitas Indonesia Fakultas Kedokteran, Community Medicine Department<br>Yuliarto, Saptadi; Brawijaya University Faculty of Medicine, Department of Pediatrics; Rumah Sakit Umum Dr Saiful Anwar, Saiful Anwar Hospital, Malang, Indonesia<br>Wati, Dyah K.; Udayana University Faculty of Medicine, Department of Child Health<br>Rayhan, Muhammad; National Cardiovascular Center, Pediatric Cardiac ICU, National Cardiovascular Center Harapan Kita, Jakarta, Indonesia<br>Valerie, Ivy C.; Udayana University Faculty of Medicine, Department of Child Health<br>Cho, Hwa; Chonnam National University Hospital, Division of Pediatric Cardiology and Pediatric Intensive Care, Department of Pediatrics, Chonnam National University Children's Hospital, Gwangju, South Korea<br>Jassat, Waasila; National Institute for Communicable Diseases<br>Blumberg, Lucille; National Institute for Communicable Diseases<br>Masha, Maureen; National Institute for Communicable Diseases<br>Semple, Calum; University of Liverpool, NIHR Health Protection Research Unit in Emerging and Zoonotic Infections, Institute of Infection Veterinary and Ecological Sciences, University of Liverpool, UK; University of Oxford, International Severe Acute Respiratory and emerging Infection Consortium (ISARIC), Pandemic Sciences Centre, University of Oxford, UK<br>Swann, Olivia; The University of Edinburgh Usher Institute of Population Health Sciences and Informatics, Centre for Medical Informatics<br>Kohns Vasconcelos, Malte; St George's University of London, Centre for Neonatal and Paediatric Infection<br>Popielska, Jolanta; Medical University of Warsaw; Warsaw's Hospital for Infectious Diseases<br>Murthy, Srinivas; The University of British Columbia, Department of Pediatrics, Division of Critical Care<br>Fowler, Robert; University of Toronto, Interdepartmental Division of Critical Care Medicine<br>Guerguerian, Anne-Marie ; The Hospital for Sick Children Department of Critical Care Medicine, Department of Critical Care Medicine, Neurosciences and Mental Health Program; University of Toronto Faculty of Medicine, Department of Critical Care Medicine

Streinu-Cercel, Anca; National Institute of Infectious Diseases Prof Dr Matei Bals
Pathmanathan, Mohan D.; Ministry of Health Malaysia, National Institutes of Health
Rojek, Amanda; University of Oxford, International Severe Acute Respiratory and emerging Infection Consortium (ISARIC), Pandemic Sciences Centre; The Royal Melbourne Hospital
Kartsonaki, Christiana; University of Oxford, International Severe Acute Respiratory and emerging Infection Consortium (ISARIC), Pandemic Sciences Centre
Gonçalves, Bronner P.; University of Oxford, International Severe Acute Respiratory and emerging Infection Consortium (ISARIC), Pandemic Sciences Centre
Citarella, Barbara; University of Oxford, International Severe Acute Respiratory and emerging Infection Consortium (ISARIC), Pandemic Sciences Centre
Merson, Laura; University of Oxford, International Severe Acute Respiratory and emerging Infection Consortium (ISARIC), Pandemic Sciences Centre
Olliaro, Piero; University of Oxford, International Severe Acute Respiratory and emerging Infection Consortium (ISARIC), Pandemic Sciences Centre
Dalton, Heidi; Inova, Inova Fairfax Medical Center

Keywords: Mortality, Neonatology, Adolescent Health, Health services research, COVID-19

SCHOLARONE™
Manuscripts

I, the Submitting Author has the right to grant and does grant on behalf of all authors of the Work (as defined in the below author licence), an exclusive licence and/or a non-exclusive licence for contributions from authors who are: i) UK Crown employees; ii) where BMJ has agreed a CC-BY licence shall apply, and/or iii) in accordance with the terms applicable for US Federal Government officers or employees acting as part of their official duties; on a worldwide, perpetual, irrevocable, royalty-free basis to BMJ Publishing Group Ltd ("BMJ") its licensees and where the relevant Journal is co-owned by BMJ to the co-owners of the Journal, to publish the Work in this journal and any other BMJ products and to exploit all rights, as set out in our licence.

The Submitting Author accepts and understands that any supply made under these terms is made by BMJ to the Submitting Author unless you are acting as an employee on behalf of your employer or a postgraduate student of an affiliated institution which is paying any applicable article publishing charge ("APC") for Open Access articles. Where the Submitting Author wishes to make the Work available on an Open Access basis (and intends to pay the relevant APC), the terms of reuse of such Open Access shall be governed by a Creative Commons licence – details of these licences and which Creative Commons licence will apply to this Work are set out in our licence referred to above.

Other than as permitted in any relevant BMJ Author's Self Archiving Policies, I confirm this Work has not been accepted for publication elsewhere, is not being considered for publication elsewhere and does not duplicate material already published. I confirm all authors consent to publication of this Work and authorise the granting of this licence.

Confidential: For Review Only

PEDIATRIC COVID-19 MORTALITY: A DATABASE ANALYSIS OF THE IMPACT OF HEALTH RESOURCE DISPARITY

First author: Eva M. Marwali[1*]

Co-authors: Aria Kekalih[2], Saptadi Yuliarto[3], Dyah K. Wati[4], Muhammad Rayhan[1], Ivy C. Valerie[4], Hwa J. Cho[5], Waasila Jassat[6], Lucille Blumberg[6], Maureen Masha[6], Malcolm G. Semple[7,8] , Olivia V. Swann[9], Malte Kohns Vasconcelos [10], Jolanta Popielska[11], Srinivas Murthy[12], Rob Fowler[13], Anne Marie Guerguerian[14], Anca Streinu-Cercel[15], Mohan D. Pathmanathan[16], Amanda Rojek[8,17], Christiana Kartsonaki[8], Bronner P. Gonçalves[8], Barbara Citarella[8], Laura Merson[8], Piero L. Olliaro[8], Heidi J. Dalton[18] and on behalf of International Severe Acute Respiratory and emerging Infection Consortium (ISARIC) Clinical Characterization Group Investigators.

Affiliation:

[1] Pediatric Cardiac ICU, National Cardiovascular Center Harapan Kita, Jakarta, Indonesia

[2] Community Medicine Department, Faculty of Medicine, Universitas Indonesia, Jakarta, Indonesia

[3] Department of Pediatrics, Faculty of Medicine, Universitas Brawijaya, Saiful Anwar Hospital, Malang, Indonesia

[4] Department of Child Health, Faculty of Medicine, Universitas Udayana/Sanglah General Hospital, Bali, Indonesia

[5] Division of Pediatric Cardiology and Pediatric Intensive Care, Department of Pediatrics, Chonnam National University Children's Hospital, Gwangju, South Korea

[6] National Institute for Communicable Diseases, Johannesburg, South Africa; Right to Care, Pretoria, South Africa

[7] NIHR Health Protection Research Unit in Emerging and Zoonotic Infections, Institute of Infection Veterinary and Ecological Sciences, University of Liverpool, UK

[8] International Severe Acute Respiratory and emerging Infection Consortium (ISARIC), Pandemic Sciences Centre, University of Oxford, UK

[9] Centre for Medical Informatics, Usher Institute, University of Edinburgh, UK

[10] Centre for Neonatal and Paediatric Infection, St. Georges, University of London, London, UK

[11] Medical University of Warsaw and Hospital for Infectious Diseases in Warsaw; Poland

Confidential: For Review Only

[12] Division of Critical Care, Department of Pediatrics, University of British Columbia, Vancouver, Canada

[13] Interdepartmental Division of Critical Care Medicine, University of Toronto, Canada

[14] Department of Critical Care Medicine, Neurosciences and Mental Health Program, The Hospital for Sick Children, University of Toronto, Toronto, Canada.

[15] National Institute for Infectious Diseases Matei Bals, Romania

[16] Institute for Clinical Research, National Institutes of Health, Ministry of Health, Malaysia

[17] Royal Melbourne Hospital, Melbourne, Australia

[18] Inova Fairfax Medical Center, Falls Church, Virginia, USA

Corresponding author:

Eva M. Marwali, MD, PhD

Pediatric Cardiac ICU, National Cardiovascular Center Harapan Kita

Jl. Let. Jend. S. Parman, Kav 87, Slipi

West Jakarta, 11420, Indonesia

Work: (+62)21 5684085 ext. 2807

Mobile: (+62)813 8570 2548

E-mail: eva.marwali@pjnhk.go.id

Abstract word count : **300**

Total word count : **2646**

Conflict of interest : none for this paper

Confidential: For Review Only

Abstract

Background: The impact of the COVID-19 pandemic on pediatric populations varied between high (HICs) versus low to middle income countries (LMICs). We sought to investigate differences in pediatric clinical outcomes and identify factors contributing to disparity between countries.

Methods: The International Severe Acute Respiratory and Emerging Infections Consortium (ISARIC) COVID-19 database was queried to include children under 19 years of age admitted to hospital from January 2020 through April 2021 with suspected or confirmed COVID-19 diagnosis. Univariate and multivariable analysis of contributing factors for mortality were assessed by country group (HICs vs. LMICs) as defined by the World Bank criteria.

Results: A total of 12,860 children (3,819 from 21 HICs and 9,041 from 15 LMICs) participated in this study. Of these, 8,961 were laboratory-confirmed and 3,899 suspected COVID-19 cases. About 52% of LMICs children were Black and more than 40% were infants and adolescent. Overall in-hospital mortality rate (95% Confidence Interval [CI]) was 3.3% [3.0 - 3.6%], higher in LMICs than HICs (4.0% [3.6 – 4.4%] and 1.7% [1.3 -2.1%], respectively). There were significant differences between country income groups in intervention profile, with higher use of antibiotics, antivirals, corticosteroids, prone positioning, high flow nasal cannula, non-invasive and invasive mechanical ventilation in HICs. Out of the 439 mechanically ventilated children, mortality occurred in 106 (24.1%) subjects, which was higher in LMICs than HICs (89 (43.6%) vs. 17 (7.2%) respectively). Pre-existing infectious comorbidities (tuberculosis and HIV) and some complications (bacterial pneumonia, acute respiratory distress syndrome, and myocarditis) were significantly higher in LMICs compared with HICs. On multivariable analysis, LMIC as country income group was associated with increased risk of mortality (adjusted HR 4.74 [3.16-7.10]).

Conclusion: Mortality and morbidities were higher in LMICs than HICs, and it may be attributable to differences in patient demographics, complications, and access to supportive and treatment modalities.

Funding: National Institute for Health Research (NIHR), UK Medical Research Council, Wellcome Trust, Department for International Development, Bill & Melinda Gates Foundation, EU Platform for European Preparedness Against (Re-)emerging Epidemics, NIHR Health Protection Research Unit (HPRU) in Emerging and Zoonotic Infections at University of Liverpool, NIHR HPRU in Respiratory Infections at Imperial College London.

Keywords: child mortality, health care rationing, health resources, SARS-CoV-2

Confidential: For Review Only

Key Messages:

What is already known?

Recent systematic reviews identified a potentially higher pediatric mortality per population from COVID-19 in LMIC compared to HIC, but concluded that heterogeneity of published studies limits firm conclusions. Correspondingly, lethality of other acute respiratory infections has been shown to be higher in LMIC.

What this study adds?

By using harmonized data collection tools of a study population of over 12,000 children, this study can directly compare inpatient management and outcomes in HIC and LMIC. Analysis finds higher mortality in LMIC although a lower proportion receive ICU admission and ventilation prior to death. Disparity in access to care and lack of available advanced medical therapies are highlighted and provide areas for collaborative efforts between clinicians, administrators and likely government groups to improve outcomes in LMIC.

How this study might affect policy?

While intensified by the pandemic, a lack of adequate resources to care for children with acute respiratory infections in LMIC is likely a general concern that requires allocation of resources. Reducing the gap in our ability to care for sick children in LMICs versus HICs will inevitably improve global outcomes during both pandemic and inter-pandemic periods.

Confidential: For Review Only

Introduction

The clinical presentation, severity, and outcomes of acute COVID-19 are different in children compared to adults. While a higher proportion of children are asymptomatic or less severely ill than in many adult reports, severe manifestations do occur.[1] While cardiac compromise in the form of Multisystem Inflammatory Syndrome in Children (MIS-C) is often described, acute respiratory distress syndrome and other organ dysfunction also occurs in children.[2,3] The risk factors for severe disease in in pediatrics are incompletely understood.[4] Furthermore, there is a lack of global data to improve understanding of the COVID-19 burden in children who live in low to middle income countries (LMICs) versus those in high-income (HICs) sites.[5]

One systematic review that summarized the difference in pediatric COVID-19 morbidity and mortality in HICs and LMICs has been published.[6] However, most studies' samples analyzed fewer than 100 patients and over half the data came from the USA and China. Furthermore, due to heterogeneous reporting of data in the included studies, the authors were limited in their ability to pool or compare data on clinical features and outcomes. A recent scoping review reported that ethnicity and socioeconomic condition were underrepresented in COVID-19 epidemiological studies.[7] We aimed to investigate the differences in survival of pediatric patients in LMICs and HICs and the factors that may contribute to such differences between regions.

Methods

Data was collected from hospitalized patients under 19 years of age with confirmed or clinically suspected COVID-19 between January 1, 2020 and March 31, 2021 admitted to institutions across the globe contributing to the International Severe Acute Respiratory and Emerging Infections Consortium (ISARIC) database according to ISARIC/ WHO Clinical Characterization Protocol for Severe Emerging Infection.[1,8] Fields for analysis were extracted from the complete dataset gathered from Research Electronic Data Capture (REDCap, version 8.11.11, Vanderbilt University, Nashville, Tennessee).[8]

Variables of interest were classified into four domains: comorbidities, presenting signs and symptoms, complications, and treatments. Comorbidity was defined as any history of pre-existing medical conditions that were not otherwise related to COVID-19 natural history and reported at admission date. Complications referred to any medical condition detected during patients' stay that was not present at admission. Therapies included were drugs, oxygen, and use of other treatments such as invasive mechanical ventilation. Ethnicity was collapsed into five categories (Black or African American, White, Asian, Mixed/others and missing/ unknown) following the Centers for Disease Control

Confidential: For Review Only

and Prevention (CDC) National Health Interview Survey (NHIS) glossary.[9] Country income groups were dichotomized into HICs and LMICs according to the latest World Bank classification.[10]

Date of admission was defined as the date of hospitalization. Descriptive statistics were described as frequencies (n) and proportions for categorical data, mean ± standard deviation (SD) or median (interquartile range [IQR]) for continuous data, and number of available data (N) for each variable. Demographic characteristics, comorbidities, complications, and treatments were compared between country income groups using Chi-square or Fischer's Exact test as indicated. Kaplan-Meier survival curves were plotted and compared using the log-rank test. Multivariable Cox proportional hazards regression models were fitted to identify mortality predictors. In hospital survival analysis was performed to obtain 28- and 90-day survival rates. Intensive care unit (ICU) admission and invasive mechanical ventilation (IMV) requirement served as proxies for morbidity. Time-to-event analyses were performed to identify morbidity and mortality-defining periods: 1) from hospital admission until ICU admission, 2) ICU admission to first intubation, 3) ICU admission to ICU discharge or death, 4) ICU discharge to hospital discharge or death. A p-value of <0.05 was considered statistically significant. Statistical analyses were performed using SPSS 25 (IBM Corp, Armonk, NY).

Results

There were 12,860 children, originating from 36 countries, 15 of which (41.7%) were LMICs and 21 (58.3%) HICs. Seventy percent (n=9041) of participants were from LMICs and 29.7% (n=3819) cases were from HICs. The majority of participants were contributed from 670 cites in South Africa (59.3%) and 346 sites in the United Kingdom (UK) (24.1%), followed by Malaysia (4.9%), Poland (2,4%), and Malawi (2.3%) (**Figure 1**). COVID-19 status was laboratory-confirmed in 69.7%; this rate was higher in HICs (73.0%) in comparison to that in LMICs (68.3%). **(Table 2)**

Adolescents aged between 12 and 17 years, were the largest age group in LMICs (25.6%), and overall (24.0%), followed by infants younger than one year of age (21%). Infants were the largest group in HICs (29.4%). Males (50.8%) and females (49.2%) were similarly represented. Black or African American participants formed more than one-third of the study population and 52.% of LMICs children (**Table 2**)

In total, 425 (3.3%) participants died. The mortality rate was higher in in LMICs compared to HICs (4% vs. 1.7%) (**Table 2**). Reported mortality in the United Kingdom was 52 children (1.2%), which does not differ significantly (p = 0.98) from the rest of HICs. Mortality in South Africa, numbering 268 children (3.5%), differed significantly

Confidential: For Review Only

(p<0.001) from 93 (6.5%) mortality in the rest of LMICs. The distribution of admission and mortality rates across the study period was presented in **Figure 2** as 3 country groups UK, South Africa and other countries. The curves showed bimodal with peaks of mortality rates coinciding with admissions during July 2020 and January 2021. Available case data in early 2020 was limited compared to later time points. In contrast to South Africa and other countries, mortality rate in the UK did not rise significantly with the first and second wave of case admissions during April-May 2020 and October 2020-January 2021.

Children in LMICs had significantly greater and earlier mortality (aHR [95% CI] 4.73 [3.16-7.10], p < 0.001). The 28 and 90-days survival among all participants were 96.7% (10,339/10,692) and 96.5% (10,642/11,024), respectively. Survival was higher at both 28 (98.3% (3049/3101)) and 90 days (98.1% (3137/3199)) in HICs than LMICs (96.0% (7290/7591) and 95% (7505/7825), respectively).

The availability of information on comorbidities varied between countries of origin. The prevalence of several comorbidities was significantly higher in HICs including chronic neurological disease, seizures, diabetes and chronic cardiac disease. Infectious diseases such as tuberculosis and HIV/AIDS were significantly higher in LMICs. (**Table 3)**

The most common presenting symptoms overall include fever (20%), cough (16.1%), and shortness of breath(10.5%). Between-group's comparisons showed that cough (HIC 14.0% vs. LMIC 26.1%), shortness of breath (9.2% vs. 16,4%) , runny nose (4.2% vs. 8.6%), loss of smell or taste (0.7% vs. 2.9%), anorexia (0.1% vs. 0.8%), and inability to walk (0.1% vs. 0.2%) were more common among patients in LMICs.

Complications during hospitalization are shown in **Table 4**. Patients in LMICs had more bacterial and cryptogenic pneumonia and ARDS, as well as other organ involvement such as brain with stroke or heart with cardiac arrest. The rates of complications were generally higher than that of HICs, except for cardiac arrhythmia and disseminated intravascular coagulation in which HICs rates were statistically higher.

The two most commonly administered therapies were antibiotics in 41.2% of participants, followed by corticosteroids in 11.4%. Antivirus was administered in 5.3% of subjects, with higher percentage observed in HIC, especially of Remdesivir which is more than twice that of LMIC. Adjunctive and supportive treatments were generally performed more often in HICs. No participants in LMICs were treated with extracorporeal membrane oxygenation, as compared to 10 participants in HICs (**Table 5**).

Participants in LMICs were most often admitted to the ICU within the first day of admission, with those who died being admitted earlier than survivors (LMICs vs. HICs: 0 [0 – 1] vs. 0 [0 – 2.5] day respectively, p = 0.03) . While

Confidential: For Review Only

time to invasive mechanical ventilation (IMV) was not significantly different for survivors vs. non-survivors in either LMIC or HICs, most received IMV in the first day of admission and all within a maximum of 3.5 days. Time from ICU admission to ICU discharge or death was significantly shorter in survivors vs. non-survivors although non survivors in HICs had longer stays than in LMICs (3 [1 – 6] vs. 9.5 [4.5–19.2] days respectively in HIC and 0 [0 – 4] vs. 4 [0.7 – 7.2] days respectively in LMIC, $p < 0.05$), Mortality was significantly higher ($p <0.001$) in LMICs compared to HICs for participants who received invasive mechanical ventilation (IMV) (43.6% vs. 7.3%) or who required ICU admission (16.7% vs. 3.5). Nearly 2.3% and 6.1% participants died without ICU and/or IMV support in LMICs in comparison to the respective 1.3% and 1.4% in HICs (**Table 6**).

Multivariable analysis was done to evaluate factors associated with mortality. Significant risk factors found were: aged <1 year (adjusted hazard ratio (aHR) [95% Confidence Interval (CI)] = 1.80 [1.01- 3.22]), low-middle income group (4.73 [3.16 - 7.10]), comorbidities such as chronic kidney disease (3.74 [2.20-6.35]) or cardiac disease (2.42 [1.50- 3.91]), and invasive mechanical ventilator requirement (3.46 [2.27 - 5.28]) or exposure to antibiotics (2.07 [1.34 - 3.22]). The use of antiviral agents (aHR= 0.55 [0.32 - 0.96]) was the only factor inversely associated with mortality (**Figure 3**).

Discussion

We present a large international cohort of children hospitalized with COVID- 19. We found that mortality was significantly higher in LMICs in comparison to HICs. Disparate care patterns were also observed, with patients in LMICs reported to receive most adjunctive and supportive therapies less frequently than patients in HICs. While these findings may represent differences in practice, they may also represent variation in available supports for children based on income status of the country. Such disparities have been described in adult COVID-19 patients, but limited data exists for children. Prior reports have focused on specific aspects of illness such as infection or cardiac dysfunction, have included small cohorts of children, or are limited to certain countries or regions.[11-20]

While the findings may be criticized as mainly representing data from 2 countries, the UK and South Africa (SA), these countries are good examples of HICs and LMICs. Statistical analysis showed no significant difference of mortality between UK and the rest of HICs. Although mortality in SA was significantly lower than the rest of LMICs, both mortalities from SA and non-SA LMICs were significantly higher than HICs group. Low number of subjects from non-SA LMICs was disproportionate to that of SA , thus conclusion can not be drawn from observed difference in mortality between them. Further, as data supplied from the UK and South Africa comes from national COVID-19

Confidential: For Review Only

research databases recruiting from a high number of sites in the UK and South Africa, and in this sense may be more representative of country income differences, as opposed to enrolment of single sites (e.g. a national referral centre) in different countries. The inclusion of children from many other countries, although relatively small cohorts form each country in comparison, does allow understanding of care patterns in areas around the world.

More participants from HICs could be admitted to ICU and received IMV than LMICs patients. While the small numbers available for analysis in some categories limit our confidence in these findings, in LMICs they do given IMV and dying within shorter periods of time than HICs. Not only were children in LMICs hospitalized with COVID-19 more likely to die, they were also shown to die earlier in their hospitalization. Despite possible confounding effects from missing data relating to severity of illness at presentation (vital signs, organ failure scores), a positive association between LMICs and mortality were consistently observed in analysis of children admitted to the ICU and those receiving IMV.

Several independent risk factors for mortality were identified in addition to country economic group. Mortality was lowest for patients aged between 1 and 5 years and higher among patients of age <1 or >5 years. This finding confirmed the U-shaped mortality pattern shown in several other reports, although infancy is not always recognized as a risk factor in small studies.[1,21,22] Comorbidities such as chronic kidney and cardiac diseases were also shown to be independent risk factors as reported by others.[19,23,24] These risk factors were more prevalent in HICs and thus did not seem to be associated with the high mortality rates noted in LMICs but this may reflect under-diagnosis possibly due to lack of diagnostic resources in some LMICs.[20] Higher rates of certain comorbidities, many infectious in nature, is another possible cause of the relationship between LMICs and mortality. Chronic respiratory failure has been associated with death in COVID-19 adult patients, with some evidence that this is occurs in pediatric cases as well. Our data also provided information on tuberculosis, which has not specifically identified as comorbidity in children with COVID in other reports. Similarly, data on the impact of HIV in children is sparse and our review finds this to be an important risk factor and more prevalent in LMICs.[23,25]

More patients receiving antiviral therapy were found in HICs vs LMICS. In fact, remdesivir, which is recommended for severe hospitalized COVID, was used in exceptionally lower percentage of LMIC subjects. We can only speculate that period of this study occurred when evidence based on antiviral efficacy was still scarce especially in children, or it may indicate lack of access to drug or lack familiarity with recommendations.[26] The recommendations for antiviral use in children with severe COVID from the National Institutes of Health (NIH) have suggested use for patients over the age of 12 years; this recommendation would not have applied to infants who had a higher risk of mortality in our study.[27,28] Further investigation of the impact of antivirals in children of all ages should be considered. The efficacy

Confidential: For Review Only

and the cost benefit of these expensive medications in resource-limited sites are needed; if valuable, improving access should then be at the core of discussions.

The use of other therapies also highlight the differences between LMICs and HICs. There was less use of many adjunct therapies associated with outcomes in adult studies such as prone positioning, high flow nasal cannula, antibiotics, steroids in LMIC sites. Whether these differences were the result of lack of availability of therapies or other regional factors cannot be determined, but it seems likely that limitation to access may influence practice. The high rate of mortality in patients outside the ICU and who died without invasive mechanical ventilation suggests that limitations to ICU beds or ventilators in LMICs likely play a large role in the excess death rates reported as compared to HICs.[29,30]

The higher prevalence of complications of respiratory disease such as ARDS, bacterial and cryptogenic organizing pneumonia (COP), and the impact of organ dysfunction outside the lung such as increased rates of myocarditis, pericarditis, endocarditis, meningitis, encephalitis, stroke and cardiac arrest observed in LMICs are also likely factors in the high death rates. Patients with MIS-C were not specifically reported in the time period of this report.

Our study has the strength of a common reporting format in participating centers around the world. We describe a relatively large number of children and are able to provide both comparisons of patient characteristics and outcomes and evaluate risk factors for outcomes using common definitions. Limitation of this study includes a predominance of patients from one LMICs and one HICs -South Africa and UK, potentially limiting the generalizability of our findings to all countries. In addition, we did not adjust for pandemic era. Inevitably we have missing data for a number of variables, including comorbidities, which limits the effective sample size of analyses examining relationships with patient characteristics and outcomes. Lack of data on nutritional status of children on each group, which may explain disparity between country income groups, was another limitation of the study. Moreover, considerable proportion of non-confirmed cases also limits the impact of this study on public health policy..

In conclusion, we found many differences in characteristics, treatments and outcomes among children from LMICs and HICs with infants had higher death rates than other children. Patients less frequently receive invasive mechanical ventilation and other supportive therapies in LMICs, which likely represents disparities in access to healthcare that influence outcomes. Reducing the gap in our ability to care for sick children in LMICs versus HICs will inevitably improve global outcomes during both pandemic and interpandemic periods.

Confidential: For Review Only

Data sharing

Data and material access are available upon data access committee agreement fulfilling the full terms (https://www.iddo.org/document/covid-19-data-access-guidelines). Applications are welcome via the Independent Data and Material Access Committee (https://isaric4c.net).

Declaration of interests

Competing interests: All authors have completed the ICMJE uniform disclosure form at www.icmje.org/coi_disclosure.pdf and declare no competing interest.

Author Contributions

The author contributions are as follows; study conception and design: EMM, AK, SY, DKW, MR, ICV, HJC, WJ, LB, MM, MGS, OVS, MKV, JP, SM, RF, AMG, AS, MDP, AR, CK, BPG, BC, LM, PLO, HD; coordination and collection of data: EMM, AK, SY, DKW, MR, ICV, HJC, WJ, LB, MM, MGS, OVS, MKV, JP, SM, RF, AMG, AS, MDP, AR, CK, BPG, BC, LM, PLO, HD; statistical analysis or interpretation of data: EMM, AK, SY, DKW, MR, ICV, HJC, WJ, LB, MM, MGS, OVS, MKV, JP, SM, RF, AMG, AS, MDP, AR, CK, BPG, BC, LM, PLO, HD; drafting the manuscript: EMM, MR, ICV; critical review and revision of the manuscript for important intellectual insight: EMM, AK, SY, DKW, MR, ICV, HJC, WJ, LB, MM, MGS, OVS, MKV, JP, SM, RF, AMG, AS, MDP, AR, CK, BPG, BC, LM, PLO, HD; study supervision: EMM, BC, LM. All authors drafted the manuscript from important intellectual viewpoints and approved the final version. Furthermore, all authors agreed to be accountable for all aspects of the work in ensuring that questions related to the accuracy or integrity of any part of the work are appropriately investigated and resolved. The corresponding author confirmed that all authors meet authorship criteria according to ICMJE.

Confidential: For Review Only

References

1. Docherty A B, Harrison E M, Green C A, Hardwick H E, Pius R, Norman L et al. Features of 20 133 UK patients in hospital with covid-19 using the ISARIC WHO Clinical Characterisation Protocol: prospective observational cohort study. BMJ 2020;369:m1985.
2. Han X, Li X, Xiao Y, Yang R, Wang Y, Wei X. Distinct Characteristics of COVID-19 Infection in Children. *Front Pediatr*. 2021;9:619738.
3. Borrelli M, Corcione A, Castellano F, Fiori Nastro F, Santamaria F. Coronavirus Disease 2019 in Children. *Front Pediatr*. 2021;9:668484.
4. Fialkowski A, Gernez Y, Arya P, Weinacht KG, Kinane TB, Yonker LM. Insight into the pediatric and adult dichotomy of COVID-19: Age-related differences in the immune response to SARS-CoV-2 infection. *Pediatr Pulmonol*. 2020 Oct;55(10):2556–2564.
5. Shadmi E, Chen Y, Dourado I, et al. Health equity and COVID-19: global perspectives. *Int J Equity Health*. 2020;19(1):104. doi:10.1186/s12939-020-01218-zKitano T, Kitano M, Krueger C, et al. The differential impact of pediatric COVID-19 between high-income countries and low- and middle-income countries: A systematic review of fatality and ICU admission in children worldwide. *PLoS ONE* 2021;16(1):e0246326.
6. Kitano T, Kitano M, Krueger C, et al. The differential impact of pediatric COVID-19 between high-income countries and low- and middle-income countries: A systematic review of fatality and ICU admission in children worldwide. *PLoS ONE* 2021;16(1):e0246326.
7. Bernardino FBS, Alencastro LC da S, Silva RA da, Ribeiro AD do N, Castilho GR de C, Gaíva MAM. Epidemiological profile of children and adolescents with COVID-19: a scoping review. *Rev bras enferm* 2021;74(Suppl 1):e20200624.
8. Abdukahil SA, Abe R, Abel L, et al. COVID-19 symptoms at hospital admission vary with age and sex: results from the ISARIC prospective multinational observational study. *Infection* 2021;49(5):889.
9. National Center for Health Statistics. National Health Interview Survey Glossary. Centers for Disease Control and Prevention.
10. The World Bank. The world by income and region. Accessed March 3, 2022. https://datatopics.worldbank.org/world-development-indicators/the-world-by-income-and-region.html.
11. Swann OV, Holden KA, Turtle A, et al. Clinical characteristics of children and young people admitted to hospital with covid-19 in United Kingdom: prospective multicentre observational cohort study. *BMJ* 2020;370:m3249.
12. Ghisolfi S, Almås I, Sandefur JC, et al. Predicted COVID-19 fatality rates based on age, sex, comorbidities and

Confidential: For Review Only

health system capacity. *BMJ Glob Health* 2020;5:e003094.

13. Ludvigsson JF. Systematic review of COVID-19 in children shows milder cases and a better prognosis than adults. *Acta Paediatr* 2020;00:1-8.

14. Chhibber-Goel J, Malhotra S, Krishnan NMA, Sharma A, et al. The profiles of first and second SARS-CoV-2 waves in the top ten COVID-19 affected countries. *J Glob Health* 2021;5:e2021082. doi:10.29392/001c.27143.

15. Delahoy MJ, Ujamaa D, Whitaker M, et al. Hospitalizations Associated with COVID-19 Among Children and Adolescents — COVID-NET, 14 States, March 1, 2020–August 14, 2021. *Morb Mortal Wkly Rep* 2021;70(36):1255–60.

16. Thelwall S, Aiano F, Harman K, et al. Risk of hospitalisation and death in children with SARS-CoV-2 delta (B.1.612.2) infection. *Lancet Child Adolesc Health* 2022. https://doi.org/10.1016/ S2352-4642(22)00096-7.

17. Liu X, Huang J, Li C, et al. The role of seasonality in the spread of COVID-19 pandemic. *Environ Res* 2021;195:110874.

18. Nachega JB, Sam-Agudu NA, Machekano RN, et al. Assessment of Clinical Outcomes Among Children and Adolescents Hospitalized With COVID-19 in 6 Sub- Saharan African Countries. *JAMA Pediatr* 2022 Mar; 176(3): e216436.

19. Gonzalez-Dambrauskas S, Vasquez-Hoyos P, Camporesi A, et al. Paediatric critical COVID-19 and mortality in a multinational prospective cohort. *The Lancet Regional Health Americas* 2022;12: 100272.

20. Oliveira EA, Colosimo EA, Silva ACS, et al. Clinical characteristics and risk factors for death among hospitalised children and adolescents with COVID-19 in Brazil: an analysis of a nationwide database. *Lancet Child Adolesc Health* 2021; 5: 559–68.

21. Khera N, Santesmasses D, Kerepesi C, et al. COVID-10 mortality rate in children is U-shaped. *Aging* 2021;13(16):19954–62.

22. O'Driscoll M, Dos Santos GB, Wang L, et al. Age-specific mortality and immunity patterns of SARS-CoV-2. *Nature* 2020. https://doi.org/10.1038/s41586-020-2918-0

23. Tsankov BK, Allaire JM, Irvine MA, et al. Severe COVID-19 Infection and Pediatric Comorbidities: A Systematic Review and Meta-Analysis. *Int J Infect Dis* 2021;103:246–56.

24. Marin BG, Aghagoli G, Lavine K, et al. Predictors of COVID-19 severity: A literature review. *Rev Med Virol* 2021;31(1):1–10.

25. Song W, Zhao J, Zhang Q, et al. COVID-19 and Tuberculosis Coinfection: An Overview of Case Reports/Case Series and Meta-Analysis. *Front Med* 2021;8:657006.

Confidential: For Review Only

26. Panda PK, Sharawat IK, Natarajan V, et al. COVID-19 treatment in children: A systematic review and meta-analysis. *J Family Med Prim Care* 2021;10:3292–302.

27. COVID-19 Treatment Guidelines Panel. Coronavirus Disease 2019 (COVID-19) Treatment Guidelines. National Institutes of Health. Available at https://www.covid19treatmentguidelines.nih.gov/. Accessed 1 Apr 2022.

28. La Tessa A, Motisi MA, Marseglia GL, et al. Use of remdesivir in children with COVID-19 infection: a quick narrative review. *Acta Biomed* 2021; 92(7): e2021524.

29. Austin S, Murthy S, Wunsch H, et al. Access to urban acute care services in high- vs. middle-income countries: an analysis of seven cities. *Intensive Care Med* 2014;40:342–352.

30. Murthy S, Leligdowicz A, Adhikari NKJ. Intensive Care Unit Capacity in Low-Income Countries: A Systematic Review. *PLoS ONE* 2015;10(1):e0116949.

Confidential: For Review Only

Figures Legend:

Figure 1. Distribution of participants by country of origin.

Figure 2. Total Covid-19 participants (bar) and mortality (line) rate trend through out data collection period.

Figure 3. Adjusted Hazard Ratio and 95% Confidence Interval of mortality risk factors in all participants.

Confidential: For Review Only

Table 1. List of participating countries and number of admitted subjects (n = 12,860)

Income group	Country name	n	%
High-income country	United Kingdom	3099	24.1
High-income country	Poland	304	2.4
High-income country	Canada	164	1.3
High-income country	Spain	87	0.7
High-income country	Germany	25	0.2
High-income country	United States	22	0.2
High-income country	Ireland	21	0.2
High-income country	France	18	0.1
High-income country	Australia	17	0.1
High-income country	Chile	16	0.1
High-income country	Israel	12	0.1
High-income country	Netherlands	11	0.1
High-income country	Italy	8	0.1
High-income country	Greece	4	0.0
High-income country	Belgium	3	0.0
High-income country	Portugal	3	0.0
High-income country	Bolivia	1	0.0
High-income country	Kuwait	1	0.0
High-income country	Norway	1	0.0
High-income country	New Zealand	1	0.0
High-income country	Saudi Arabia	1	0.0
Lower-middle income country	South Africa	7621	59.3
Lower-middle income country	Malaysia	627	4.9
Lower-middle income country	Malawi	296	2.3
Lower-middle income country	Romania	135	1.0
Lower-middle income country	Colombia	112	0.9
Lower-middle income country	Indonesia	73	0.6
Lower-middle income country	Peru	49	0.4
Lower-middle income country	Pakistan	35	0.3
Lower-middle income country	India	24	0.2
Lower-middle income country	Honduras	21	0.2
Lower-middle income country	Argentina	18	0.1
Lower-middle income country	Mexico	11	0.1
Lower-middle income country	Brazil	10	0.1
Lower-middle income country	Nepal	6	0.0
Lower-middle income country	Russia	3	0.0

Confidential: For Review Only

Table 2. Baseline characteristics of all study participants (n = 12,860)

		Country Income						p
		HICs (N=3819)		LMICs (N=9041)		Total		
		n	%	n	%	n	%	
Age*	<1 year	1124	29.4	1574	17.4	2698	21.0	<0.001
	1 to <5 years	707	18.5	1887	20.9	2594	20.2	
	5 to <12 years	796	20.8	1621	17.9	2417	18.8	
	12 to <17 years	770	20.2	2311	25.6	3081	24.0	
	≥17 years	422	11.1	1648	18.2	2070	16.1	
Sex*	Male	2026	53.1	4497	49.8	6523	50.8	0.002
	Female	1788	46.9	4537	50.2	6325	49.2	
Ethnicity *	Black or African American	223	5.8	4747	52.5	4970	38.6	<0.001
	Missing and unknown	1221	32.0	3509	38.8	4730	36.8	
	White	1624	42.5	186	2.1	1810	14.1	
	Asian	561	14.7	168	1.9	729	5.7	
	Mixed and others	190	5.0	431	4.8	621	4.8	
COVID-19 status*	Confirmed	2786	73	6175	68.3	8961	69.7	<0.001
	Suspect	1033	27	2866	31.7	3899	30.3	
Outcome*	Discharged	3423	89.6	7967	88.1	11390	88.6	<0.001
	Still in hospital	140	3.7	357	3.9	497	3.9	
	Transferred	192	5.0	356	3.9	548	4.3	
	Death	64	1.7	361	4.0	425	3.3	

Significant with p-value <0.05 using Chi-square test (*).

HICs: high-income countries, LMICs : low- to middle-income countries.

Confidential: For Review Only

Table 3. Comparison of comorbidities between country income groups

	Country Income									p
	HICs			LMICs			Total			
	N	n	%	N	n	%	N	n	%	
Chronic neurological disorder*	2588	191	7.4	902	14	1.6	3490	205	5.9	0.001
Seizure*	2490	111	4.5	899	16	1.8	3389	127	3.7	0.001
Smoking	2091	82	3.9	1509	46	3.0	3600	128	3.6	0.215
Diabetes*	2592	95	3.7	4468	149	3.3	7060	244	3.5	0.001
Chronic cardiac disease*	2587	141	5.5	3578	48	1.3	6165	189	3.1	0.001
Obesity*	2438	90	3.7	1736	39	2.2	4174	129	3.1	0.001
Tuberculosis*	290	-	-	3649	102	2.8	3939	102	2.6	0.001
Chronic hematological disease*	2475	77	3.1	899	9	1.0	3374	86	2.5	0.001
HIV/AIDS*	2562	1	<0.01	4382	142	3.2	6944	143	2.1	0.001
Rare diseases and inborn errors of metabolism	1823	36	2.0	0	-	-	1823	36	2.0%	0.715
Malnutrition	2539	35	1.4	901	26	2.9%	3440	61	1.8%	0.412
Malignant neoplasm*	2571	64	2.5	4164	27	0.6%	6735	91	1.4%	0.001
Rheumatological disorder*	2491	45	1.8	898	2	0.2%	3389	47	1.4%	0.001
Chronic kidney disease*	2596	59	2.3	4147	28	0.7	6743	87	1.3	0.001
Liver disease	2623	12	0.5	902	-	-	3525	12	0.3	0.866

Significant with p-value <0.05 using Chi-square test (*).

HICs: high-income countries; LMICs : low- to middle-income countries; N= denominators; n= total patients with comorbidities.

Confidential: For Review Only

Table 4. Comparison of complications between country income groups

	Country Income									
	HICs			LMICs			Total			p
	N	n	%	N	n	%	N	n	%	
Bacterial pneumonia*	3497	115	3.3	1107	102	9.2	4604	217	4.7	<0.001
ARDS*	3153	31	1.0	4972	267	5.4	8125	298	3.7	<0.001
AKI	3624	110	3.0	1410	49	3.5	5034	159	3.2	0.477
Seizure	3627	103	2.8	1412	39	2.8	5039	142	2.8	0.958
Pleural effusion	3511	82	2.3	1110	36	3.2	4621	118	2.6	0.118
Myocarditis and pericarditis*	2858	42	1.5	427	22	5.2	3285	64	1.9	<0.001
Bronchiolitis	3620	57	1.6	1412	21	1.5	5032	78	1.6	0.922
Cardiac arrhythmia*	3635	70	1.9	1407	13	0.9	5042	83	1.6	0.017
Endocarditis*	692	5	0.7	487	14	2.9	1179	19	1.6	0.008
Cardiac arrest*	3535	19	0.5	1412	55	3.9	4947	74	1.5	<0.001
DIC*	3498	48	1.3	4668	58	1.2	8166	104	1.3	0.850
Meningitis and encephalitis*	3621	16	0.4	1413	20	1.4	5034	36	0.7	<0.001
Pneumothorax	3530	15	0.4	1109	9	0.8	4639	24	0.5	0.185
Stroke/cerebrovascular complication[a]	3527	7	0.2	1109	11	1.0	4636	18	0.4	0.001
Pulmonary Embolism	1928	4	0.2	293	2	0.7	2221	6	0.3	0.182
Cardiac ischemia	3510	7	0.2	1110	4	0.4	4620	11	0.2	0.309
Myocardial infarction	312	1	0.3	298	-	-	610	1	0.2	1.000
Sepsis	-	-	-	3862	8	0.2	3862	8	0.2	-
DVT	1606	1	0.1	3	-	-	1609	1	0.1	1.000
COP[a]	3398	-	-	1110	5	0.5	4508	5	0.1	0.001

Significant with p-value <0.05 using Chi-square test (*) or Fischer's Exact test ([a])

AKI: acute kidney injury; ARDS: acute respiratory distress syndrome; COP: cryptogenic organizing pneumonia; DIC: disseminated intravascular coagulation; DVT: deep vein thrombosis; HICs: high-income countries; LMICs : low- to middle-income countries; N= denominators; n= total patients with complications.

Confidential: For Review Only

Table 5. Treatment profile of study participants.

	Country Income									
	HICs			LMICs			Total			p
	N	n	%	N	n	%	N	n	%	
Antibiotics*	3685	2189	59.4	4349	1122	25.8	8034	3311	41.2	<0.001
Corticosteroid*	3663	564	15.4	4905	417	8.5	8568	981	11.4	<0.001
HFNC*	3380	282	8.3	3967	147	3.7	7347	429	5.8	<0.001
IMV*	3620	235	6.5	4684	204	4.3	8304	439	5.3	<0.001
Antivirus*	3672	271	7.4	4341	172	4.0	8013	443	5.3	<0.001
Remdesivir	3672	96	2.6	4341	17	0.4	8013	113	1.4	
Neuraminidase inhibitor	3672	32	0.9	4341	23	0.5	8013	113	55	
Inotropic/vasopressor*	3451	201	5.8	4961	174	3.5	8412	375	4.4	<0.001
Prone positioning*	3532	64	1.8	4647	39	0.8	8179	103	1.2	<0.001
Anticoagulant	3819	50	1.3	9041	88	1.0	12860	138	1.1	0.091
NIV*	3627	53	1.5	4966	17	0.3	8593	70	0.8	<0.001
RRT	3573	19	0.5	4334	27	0.6	7907	46	0.6	0.084
ECMOa	3608	10	0.3	1086	-	-	4694	10	0.2	<0.001

Significant with p-value <0.05 using Chi-square test (*) or Fischer's Exact test ([a])
ECMO: extracorporeal membrane oxygenation; HFNC: high-flow nasal cannula; IMV: invasive mechanical ventilation; NIV: non-invasive ventilation; RRT: renal replacement therapy; HICs: high-income countries; LMICs : low- to middle-income countries; N= denominators; n= total patients with treatments.

Table 6. Mortality based on ICU admission and invasive mechanical ventilation use.

		Mortality based on Country Income								
		HICs			LMICs			Total		
		N	n	%	N	n	%	N	n	%
ICU*	Yes	679	24	3.5	1037	173	16.7	1716	197	11.5
	No	3140	40	1.3	8004	188	2.3	11144	228	2.0
IMV*	Yes	235	17	7.2	204	89	43.6	439	106	24.1
	No	3385	47	1.4	4480	272	6.1	7865	319	4.0

Significant with p-value <0.05 using Chi-square test (*)
HICs: high-income countries; LMICs: low- to middle-income countries; ICU: intensive care unit; IMV: invasive mechanical ventilation; N= denominator (total patients with or without ICU/ IMV); n= total mortality.

Confidential: For Review Only

ISARIC Clinical Characterisation Group

Sheryl Ann Abdukahil, Nurul Najmee Abdulkadir, Laurent Abel, Lara Absil, Subhash Acharya, Diana Adrião, Younes Ait Tamlihat, Ernita Akmal, Eman Al Qasim, Tala Al-dabbous, Beatrice Alex, Kévin Alexandre, Huda Alfoudri, Kazali Enagnon Alidjnou, Clotilde Allavena, Nathalie Allou, Rita Alves, Joana Alves Cabrita, Maria Amaral, Nur Amira, Phoebe Ampaw, Claire Andréjak, Andrea Angheben, François Angoulvant, Séverine Ansart, Sivanesen Anthonidass, Carlos Alexandre Antunes de Brito, Ardiyan Apriyana, Yaseen Arabi, Carolline Araujo, Patrick Archambault, Jean-Benoît Arlet, Christel Arnold-Day, Elise Artaud-Macari, Diptesh Aryal, Muhammad Ashraf, Jean Baptiste Assie, Amirul Asyraf, Anika Atique, AM Udara Lakshan Attanyake, Johann Auchabie, Hugues Aumaitre, Adrien Auvet, Laurène Azemar, Cecile Azoulay, Benjamin Bach, Delphine Bachelet, Claudine Badr, J. Kenneth Baillie, Nazreen Abu Bakar, Mohanaprasanth Balakrishnan, Valeria Balan, Firouzé Bani-Sadr, Renata Barbalho, Wendy S. Barclay, Saef Umar Barnett, Audrey Barrelet, Marie Bartoli, Joaquín Baruch, Romain Basmaci, Muhammad Fadhli Hassin Basri, Jules Bauer, Diego Fernando Bautista Rincon, Abigail Beane, Alexandra Bedossa, Ker Hong Bee, Husna Begum, Sylvie Behilill, Albertus Beishuizen, Anna Beltrame, Marine Beluze, Nicolas Benech, Lionel Eric Benjiman, Dehbia Benkerrou, Delphine Bergeaud, José Luis Bernal Sobrino, Giulia Bertoli, Simon Bessis, Sybille Bevilcaqua, Karine Bezulier, Nowneet Kumar Bhat, Krishna Bhavsar, Farah Nadiah Bidin, Felwa Bin Humaid, Mohd Nazlin Bin Kamarudin, François Bissuel, Laurent Bitker, Jonathan Bitton, Mathieu Blot, Lucille Blumberg, Laetitia Bodenes, Debby Bogaert, Anne-Hélène Boivin, Isabela Bolaños, Pierre-Adrien Bolze, François Bompart, Diogo Borges, Raphaël Borie, Hans Martin Bosse, Elisabeth Botelho-Nevers, Lila Bouadma, Olivier Bouchaud, Sabelline Bouchez, Dounia Bouhmani, Damien Bouhour, Kévin Bouiller, Laurence Bouillet, Camile Bouisse, Anne-Sophie Boureau, Maude Bouscambert, Aurore Bousquet, Jason Bouziotis, Bianca Boxma, Marielle Boyer-Besseyre, Fernando Augusto Bozza, Axelle Braconnier, Cynthia Braga, Timo Brandenburger, Kathy Brickell, Marjolein Brusse-Keizer, Polina Bugaeva, Marielle Buisson, Erlina Burhan, Aidan Burrell, Ingrid G. Bustos, Denis Butnaru, André Cabie, Eder Caceres, Cyril Cadoz, Rui Caetano Garcês, Jose Andres Calvache, Valentine Campana, Paul Campbell, Pauline Caraux-Paz, Chiara Simona Cardellino, Sofia Cardoso, Filipe Cardoso, Filipa Cardoso, Nicolas Carlier, Thierry Carmoi, Marie-Christine Carret, François Martin Carrier, Gail Carson, Maire-Laure Casanova, Mariana Cascão, José Casimiro, Nidyanara Castanheira, Guylaine Castor-Alexandre, François-Xavier Catherine, Paolo Cattaneo, Roberta Cavalin, Minerva Cervantes-Gonzalez, Anissa Chair, Catherine Chakveatze, Adrienne Chan, Meera Chand, Christelle Chantalat Auger, Jean-Marc Chapplain, Julie Chas, Anjellica Chen, Matthew Pellan Cheng, Antoine Cheret, Thibault Chiarabini, Julian Chica, Suresh Kumar Chidambaram, Leong Chin Tho, Catherine Chirouze, Bernard Cholley, Marie-Charlotte Chopin, Ting Soo Chow, Hiu Jian Chua, Jonathan Chua, Barbara Wanjiru Citarella, Emma Clarke, Sara Clohisey, Alexandra Coelho, Megan Coles, Gwenhaël Colin, Pamela Combs, Marie Connor, Anne Conrad, Graham S. Cooke, Mary Copland, Hugues Cordel, Amanda Corley, Sabine Cornelis, Alexander Daniel Cornet, Arianne Joy Corpuz, Grégory Corvaisier, Emma Costigan, Camille Couffignal, Sandrine Couffin-Cadiergues, Roxane Courtois, Stéphanie Cousse, Rachel Cregan, Sabine Croonen, Claudina Cruz, Juan Luis Cruz Bermúdez, Jaime Cruz Rojo, Elodie Curlier, Paula Custodio, Ana da Silva Filipe, Charlene Da Silveira, Andrew Dagens, Heidi Dalton, Jo Dalton, Juliana Damas, Nick Daneman, Corinne Daniel, Emmanuelle A Dankwa, Jorge Dantas, Etienne De Montmollin, Rafael Freitas de Oliveira França, Thushan de Silva, Peter de Vries, Jillian Deacon, Alexa Debard, Marie-Pierre Debray, Nathalie DeCastro, William Dechert, Lauren Deconninck, Romain Decours, Eve Defous, Isabelle Delacroix, Eric Delaveuve, Karen Delavigne, Christelle Delmas, Pierre Delobel, Corine Delsing, Elisa Demonchy, Emmanuelle Denis, Dominique Deplanque, Pieter Depuydt, Diane Descamps, Mathilde Desvallées, Santi Dewayanti, Pathik Dhanger, Alpha Diallo, Sylvain Diamantis, André Dias, Rodrigo Diaz, Kévin Didier, Jean-Luc Diehl, Wim Dieperink, Jérôme Dimet, Vincent Dinot, Fara Diop, Alphonsine Diouf, Yael Dishon, Félix Djossou, Annemarie B. Docherty, Arjen M Dondorp, Maria Donnelly, Christl A. Donnelly, Chloe Donohue, Céline Dorival, Eric D'Ortenzio, James Joshua Douglas, Nathalie Dournon, Tom Drake, Murray Dryden, Murray Dryden, Claudio Duarte Fonseca, Vincent Dubee, François Dubos, Alexandre Ducancelle, Paul Dunand, Jake Dunning, Bertrand Dussol, Xavier Duval, Sim Choon Ean, Mohammed El Sanharawi, Brigitte Elharrar, Philippine Eloy, Isabelle Enderle, Chan Chee Eng, Ilka Engelmann, Vincent Enouf, Olivier Epaulard, Martina Escher, Hélène Esperou, Catarina Espírito Santo, Marina Esposito-Farese, João Estevão, Manuel

Confidential: For Review Only

Etienne, Nadia Ettalhaoui, Mirjam Evers, Marc Fabre, Isabelle Fabre, Amna Faheem, Arabella Fahy, Cameron J. Fairfield, Pedro Faria, Ahmed Farooq, Hanan Fateena, Karine Faure, Raphaël Favory, Jorge Fernandes, Marília Andreia Fernandes, François-Xavier Ferrand, Eglantine Ferrand Devouge, Joana Ferrão, Mário Ferraz, Bernardo Ferreira, Nicolas Ferriere, Céline Ficko, Claudia Figueiredo-Mello, Thomas Flament, Clara Flateau, Tom Fletcher, Victor Fomin, Tatiana Fonseca, Patricia Fontela, Simon Forsyth, Erwan Fourn, Robert A. Fowler, John F Fraser, Christophe Fraser, Marcela Vieira Freire, Ana Freitas Ribeiro, Stéphanie Fry, Valérie Gaborieau, Rostane Gaci, Jean-Charles Gagnard, Amandine Gagneux-Brunon, Carrol Gamble, Yasmin Gani, Noelia García Barrio, Esteban Garcia-Gallo, Denis Garot, Valérie Garrait, Nathalie Gault, Alexandre Gaymard, Eva Geraud, Louis Gerbaud Morlaes, Nuno Germano, Jade Ghosn, Carlo Giaquinto, Jess Gibson, Tristan Gigante, Morgane Gilg, Guillermo Giordano, Michelle Girvan, Valérie Gissot, Daniel Glikman, Petr Glybochko, Geraldine Goco, François Goehringer, Siri Goepel, Jean-Christophe Goffard, Jin Yi Goh, Marie Gominet, Bronner P. Gonçalves, Patricia Gordon, Isabelle Gorenne, Laure Goubert, Cécile Goujard, Tiphaine Goulenok, Pascal Granier, Christopher A. Green, Courtney Greene, William Greenhalf, Segolène Greffe, Fiona Griffiths, Albert Groenendijk, Anja Grosse Lordemann, Heidi Gruner, Yusing Gu, Jérémie Guedj, Martin Guego, Dewi Guellec, Anne-Marie Guerguerian, Daniela Guerreiro, Romain Guery, Anne Guillaumot, Laurent Guilleminault, Thomas Guimard, Marieke Haalboom, Daniel Haber, Hannah Habraken, Ali Hachemi, Nadir Hadri, Fakhir Haidri, Matthew Hall, Sophie Halpin, Ansley Hamer, Rebecca Hamidfar, Lim Yuen Han, Rashan Haniffa, Kok Wei Hao, Hayley Hardwick, Ewen M. Harrison, Janet Harrison, Lars Heggelund, Ross Hendry, Maxime Hentzien, Diana Hernandez, Astarini Hidayah, Rupert Higgins, Samuel Hinton, Hikombo Hitoto, Antonia Ho, Yi Bin Ho, Alexandre Hoctin, Isabelle Hoffmann, Wei Han Hoh, Peter Horby, Ikram Houas, Catherine L. Hough, Jimmy Ming-Yang Hsu, Jean-Sébastien Hulot, Samreen Ijaz, Arfan Ikram, Hajnal-Gabriela Illes, Patrick Imbert, Hugo Inácio, Yun Sii Ing, Elias Iosifidis, Sarah Isgett, Tiago Isidoro, Nadiah Ismail, Margaux Isnard, Danielle Jaafar, Salma Jaafoura, Julien Jabot, Clare Jackson, Nina Jamieson, Pierre Jaquet, Waasila Jassat, Coline Jaud-Fischer, Stéphane Jaureguiberry, Florence Jego, Anilawati Mat Jelani, Ong Yiaw Joe, Mark Joseph, Cédric Joseph, Swosti Joshi, Mercé Jourdain, Philippe Jouvet, Dafsah Juzar, Ouifiya Kafif, Florentia Kaguelidou, Neerusha Kaisbain, Thavamany Kaleesvran, Sabina Kali, Muhammad Aisar Ayadi Kamaluddin, Zul Amali Che Kamaruddin, Nadiah Kamarudin, Kong Yeow Kang, Pratap Karpayah, Todd Karsies, Christiana Kartsonaki, Anant Kataria, Kevin Katz, Seán Keating, Yvelynne Kelly, Sadie Kelly, Kalynn Kennon, Younes Kerroumi, Sharma Keshav, Antoine Khalil, Coralie Khan, Irfan Khan, Quratul Ain Khan, Sushil Khanal, Abid Khatak, Saye Khoo, Ryan Khoo, Denisa Khoo, Khor How Kiat, Peter Kiiza, Antoine Kimmoun, Detlef Kindgen-Milles, Paul Klenerman, Rob Klont, Gry Kloumann Bekken, Stephen R Knight, Robin Kobbe, Chamira Kodippily, Malte Kohns Vasconcelos, Arsène Kpangon, Vinothini Krishnan, Ganesh Kumar, Bharath Kumar Tirupakuzhi Vijayaraghavan, Pavan Kumar Vecham, Vinod Kumar, Neurinda Permata Kusumastuti, Demetrios Kutsogiannis, Marie Lachatre, Marie Lacoste, Marie Lagrange, Fabrice Laine, Olivier Lairez, Antonio Lalueza, Marc Lambert, Marie Langelot-Richard, Vincent Langlois, Marina Lanza, Cédric Laouénan, Samira Laribi, Delphine Lariviere, Stéphane Lasry, Odile Launay, Didier Laureillard, Yoan Lavie-Badie, Andy Law, Teresa Lawrence, Minh Le, Clément Le Bihan, Cyril Le Bris, Georges Le Falher, Lucie Le Fevre, Quentin Le Hingrat, Marion Le Maréchal, Soizic Le Mestre, Gwenaël Le Moal, Vincent Le Moing, Hervé Le Nagard, Paul Le Turnier, Ema Leal, Marta Leal Santos, Todd C. Lee, James Lee, Jennifer Lee, Heng Gee Lee, Biing Horng Lee, Yi Lin Lee, Gary Leeming, Laurent Lefebvre, Bénédicte Lefebvre, Benjamin Lefèvre, Sylvie LeGac, Jean-Daniel Lelievre, François Lellouche, Adrien Lemaignen, Véronique Lemee, Anthony Lemeur, Ha Sha Lene, Jenny Lennon, Marc Leone, Quentin Lepiller, François-Xavier Lescure, Olivier Lesens, Mathieu Lesouhaitier, Yves Levy, Bruno Levy, Claire Levy-Marchal, Erwan L'Her, Gianluigi Li Bassi, Geoffrey Liegeon, Wei Shen Lim, Kah Chuan Lim, Bruno Lina, Lim Lina, Guillaume Lingas, Sylvie Lion-Daolio, Samantha Lissauer, Marine Livrozet, Patricia Lizotte, Navy Lolong, Leong Chee Loon, Diogo Lopes, Anthony L. Loschner, Paul Loubet, Bouchra Loufti, Guillame Louis, Lee Lee Low, Marije Lowik, Jia Shyi Loy, Jean Christophe Lucet, Carlos Lumbreras Bermejo, Liem Luong, Dominique Luton, Ruth Lyons, Oryane Mabiala, Sara Machado, Moïse Machado, Gabriel Macheda, Hashmi Madiha, Guillermo Maestro de la Calle, Rafael Mahieu, Sophie Mahy, Mylène Maillet, Thomas Maitre, Nadia Malik, Fernando Maltez, Denis Malvy, Victoria Manda, Jose M. Mandei, Laurent Mandelbrot, Julie Mankikian, Edmund Manning, Aldric Manuel, Ceila Maria Sant`Ana Malaque, Daniel Marino, Samuel Markowicz, Laura Marsh, John Marshall, Celina Turchi Martelli, Guillaume Martin-Blondel, Martin Martinot, Ana Martins, Caroline Martins Rego, Eva Miranda Marwali, Marsilla

Confidential: For Review Only

Marzukie, David Maslove, Sabina Mason, Sobia Masood, Basri Mat Nor, Moshe Matan, Daniel Mathieu, Mathieu Mattei, Romans Matulevics, Laurence Maulin, Thierry Mazzoni, Colin McArthur, Anne McCarthy, Sarah E. McDonald, Kenneth A. McLean, Paul McNally, Cécile Mear-Passard, Ogechukwu Menkiti, Kusum Menon, France Mentré, Alexander J. Mentzer, Noémie Mercier, Emmanuelle Mercier, Antoine Merckx, Mayka Mergeay-Fabre, Laura Merson, António Mesquita, Agnès Meybeck, Alison M. Meynert, Vanina Meysonnier, Amina Meziane, Mehdi Mezidi, Céline Michelanglei, Isabelle Michelet, Nor Arisah Misnan, Tahira Jamal Mohamed, Nik Nur Eliza Mohamed, Asma Moin, Agostinho Monteiro, Claudia Montes, Shona C. Moore, Sarah Moore, Lina Morales Cely, Lucia Moro, Ben Morton, Hugo Mouquet, Clara Mouton Perrot, Julien Moyet, Caroline Mudara, Ng Yong Muh, Dzawani Muhamad, Jimmy Mullaert, Karl Erik Müller, Daniel Munblit, Marlène Murris, Srinivas Murthy, Himed Musaab, Himasha Muvindi, Gugapriyaa Muyandy, Nadège Neant, Nikita Nekliudov, Raul Neto, Anthony Nghi, Duc Nguyen, Alistair Nichol, Nurul Amani Mohd Noordin, Marion Noret, Nurul Faten Izzati Norharizam, Lisa Norman, Mahdad Noursadeghi, Karolina Nowicka, Saad Nseir, Nurnaningsih Nurnaningsih, Elsa Nyamankolly, Annmarie O'Callaghan, Katie O'Hearn, Agnieszka Oldakowska, Piero L. Olliaro, David S.Y. Ong, Jee Yan Ong, Wilna Oosthuyzen, Peter Openshaw, Saijad Orakzai, Claudia Milena Orozco-Chamorro, Linda O'Shea, Siti Zubaidah Othman, Nadia Ouamara, Rachida Ouissa, Eric Oziol, Maïder Pagadoy, Justine Pages, Massimo Palmarini, Prasan Kumar Panda, Lai Hui Pang, Nathalie Pansu, Aurélie Papadopoulos, Rachael Parke, Melissa Parker, Jérémie Pasquier, Bruno Pastene, Mohan Dass Pathmanathan, Juliette Patrier, Mical Paul, Christelle Paul, Jorge Paulos, William A. Paxton, Jean-François Payen, Kalaiarasu Peariasamy, Miguel Pedrera Jiménez, Florent Peelman, Nathan Peiffer-Smadja, Vincent Peigne, Ithan D. Peltan, Rui Pereira, Daniel Perez, Thomas Perpoint, Vincent Pestre, Michele Petrovic, Ventzislava Petrov-Sanchez, Gilles Peytavin, Scott Pharand, Walter Picard, Olivier Picone, Carola Pierobon, Djura Piersma, Carlos Pimentel, Raquel Pinto, Catarina Pires, Isabelle Pironneau, Lionel Piroth, Riinu Pius, Laurent Plantier, Hon Shen Png, Julien Poissy, Ryadh Pokeerbux, Maria Pokorska-Spiewak, Georgios Pollakis, Diane Ponscarme, Jolanta Popielska, Douwe F. Postma, Diana Póvoas, Sébastien Preau, Jean-Charles Preiser, Mark G. Pritchard, Gamage Dona Dilanthi Priyadarshani, Oriane Puéchal, Vilmaris Quinones-Cardona, Víctor Quirós González, Mohammed Quraishi, Christian Rabaud, Marie Rafiq, Rozanah Abd Rahman, Ahmad Kashfi Haji Ab Rahman, Giri Shan Rajahram, Nagarajan Ramakrishnan, José Ramalho, Ahmad Afiq Ramli, Blandine Rammaert, Grazielle Viana Ramos, Christophe Rapp, Aasiyah Rashan, Thalha Rashan, Menaldi Rasmin, Cornelius Rau, Tharmini Ravi, Stanislas Rebaudet, Sarah Redl, Brenda Reeve, Attaur Rehman, Jonathan Remppis, Martine Remy, Hongru Ren, Hanna Renk, Anne-Sophie Resseguier, Matthieu Revest, Oleksa Rewa, Luis Felipe Reyes, David Richardson, Laurent Richier, Siti Nurul Atikah Ahmad Ridzuan, Ana L Rios, Asgar Rishu, Patrick Rispal, Karine Risso, Stephanie Roberts, David L. Robertson, Olivier Robineau, Paola Rodari, Pierre-Marie Roger, Emmanuel Roilides, Amanda Rojek, Juliette Romaru, Mélanie Roriz, Manuel Rosa-Calatrava, Michael Rose, Andrea Rossanese, Bénédicte Rossignol, Patrick Rossignol, Stella Rousset, Carine Roy, Benoît Roze, Desy Rusmawatiningtyas, Clark D. Russell, Musharaf Sadat, Valla Sahraei, Maximilien Saint-Gilles, Stéphane Sallaberry, Charlotte Salmon Gandonniere, Hélène Salvator, Olivier Sanchez, Vanessa Sancho-Shimizu, Zulfiqar Sandhu, Pierre-François Sandrine, Oana Sandulescu, Shirley Sarfo-Mensah, Benjamine Sarton, Egle Saviciute, Parthena Savvidou, Yen Tsen Saw, Arnaud Scherpereel, Marion Schneider, Janet T. Scott, James Scott-Brown, Nicholas Sedillot, Jaganathan Selvanayagam, Mageswari Selvarajoo, Caroline Semaille, Malcolm G. Semple, Rasidah Bt Senian, Eric Senneville, Tânia Sequeira, Ary Serpa Neto, Pablo Serrano Balazote, Ellen Shadowitz, Syamin Asyraf Shahidan, Mohammad Shamsah, Shaikh Sharjeel, Catherine A. Shaw, Victoria Shaw, Ashraf Sheharyar, Mohiuddin Shiekh, Sally Shrapnel, Nassima Si Mohammed, Ng Yong Siang, Jeanne Sibiude, Louise Sigfrid, Benedict Sim Lim Heng, Karisha Sivam, Sue Smith, Morgane Snacken, Tze Vee Soh, Joshua Solomon, Tom Solomon, Agnès Sommet, Rima Song, Tae Song, Azlan Mat Soom, Albert Sotto, Edouard Soum, B. P. Sanka Ruwan Sri Darshana, Shiranee Sriskandan, Sarah Stabler, Ymkje Stienstra, Adrian Streinu-Cercel, Anca Streinu-Cercel, David Stuart, Jacky Y. Suen, Charlotte Summers, Deepashankari Suppiah, Andrey Svistunov, Sarah Syahrin, Jaques Sztajnbok, Shirin Tabrizi, Fabio S. Taccone, Lysa Tagherset, Shahdattul Mawarni Taib, Ewa Talarek, Kim Keat Tan, Yan Chyi Tan, Coralie Tardivon, Pierre Tattevin, M Azhari Taufik, Richard S. Tedder, Tze Yuan Tee, João Teixeira, Marie-Capucine Tellier, Sze Kye Teoh, François Téoulé, Olivier Terrier, Nicolas Terzi, Hubert Tessier-Grenier, Alif Adlan Mohd Thabit, Zhang Duan Tham, Suvintheran Thangavelu, Vincent Thibault, Simon-Djamel Thiberville, Benoît Thill, Jananee Thirumanickam, David Thomson, Emma C. Thomson, Surain Raaj Thanga

Confidential: For Review Only

Thurai, Ryan S. Thwaites, Paul Tierney, Peter S Timashev, Jean-François Timsit, Noémie Tissot, Jordan Zhien Yang Toh, Sia Loong Tonnii, Marta Torre, Margarida Torres, Tony Trapani, Théo Treoux, Cécile Tromeur, Tiffany Trouillon, Jeanne Truong, Christelle Tual, Sarah Tubiana, Jean-Marie Turmel, Lance C.W. Turtle, PG Ishara Udayanga, Andrew Udy, Timothy M. Uyeki, Luís Val-Flores, Amélie Valran, Marcel van den Berge, Job van der Palen, Paul van der Valk, Peter Van der Voort, Sylvie Van Der Werf, Jarne Van Hattem, Carolien van Netten, Ilonka van Veen, Noémie Vanel, Shoban Raj Vasudayan, Charline Vauchy, Shaminee Veeran, Aurélie Veislinger, Annelies Verbon, James Vickers, José Ernesto Vidal, César Vieira, Benoit Visseaux, Harald Vonkeman, Fanny Vuotto, Noor Hidayu Wahab, Suhaila Abdul Wahab, Nadirah Abdul Wahid, Steve Webb, Jia Wei, Katharina Weil, Tan Pei Wen, Sanne Wesselius, Murray Wham, Nicole White, Paul Henri Wicky, Aurélie Wiedemann, Evert-Jan Wils, Calvin Wong, Xin Ci Wong, Yew Sing Wong, Teck Fung Wong, Gan Ee Xian, Lim Saio Xian, Kuan Pei Xuan, Siti Rohani Binti Mohd Yakop, Yazdan Yazdanpanah, Nicholas Yee Liang Hing, Cécile Yelnik, Chian Hui Yeoh, Stephanie Yerkovich, Hodane Yonis, Obada Yousif, Saptadi Yuliarto, Marion Zabbe, Masliza Zahid, Maram Zahran, Nor Zaila Binti Zaidan, Maria Zambon, Konrad Zawadka, Nurul Zaynah, Hiba Zayyad, David Zucman, Mazankowski Heart Institute.

Confidential: For Review Only

Confidential: For Review Only

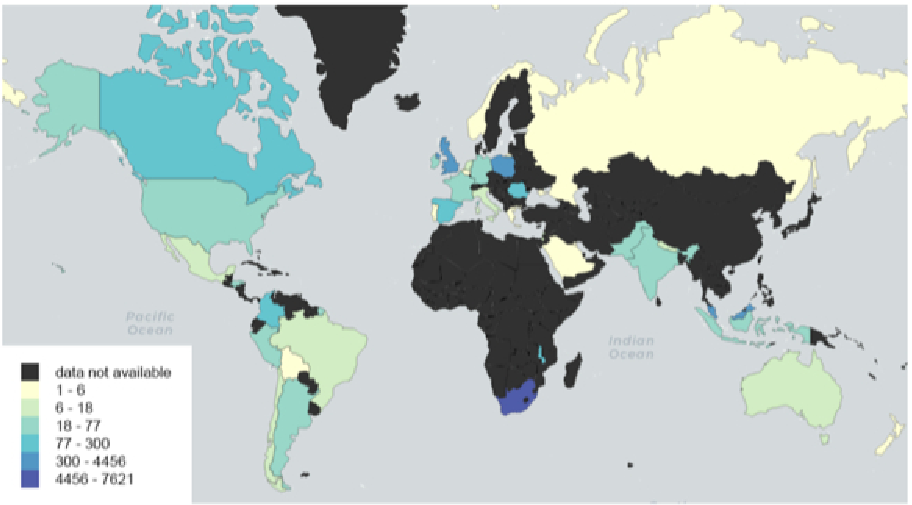

Figure 1. Distribution of participants by country of origin

Distribution of participants by country of origin.

108x65mm (150 x 150 DPI)

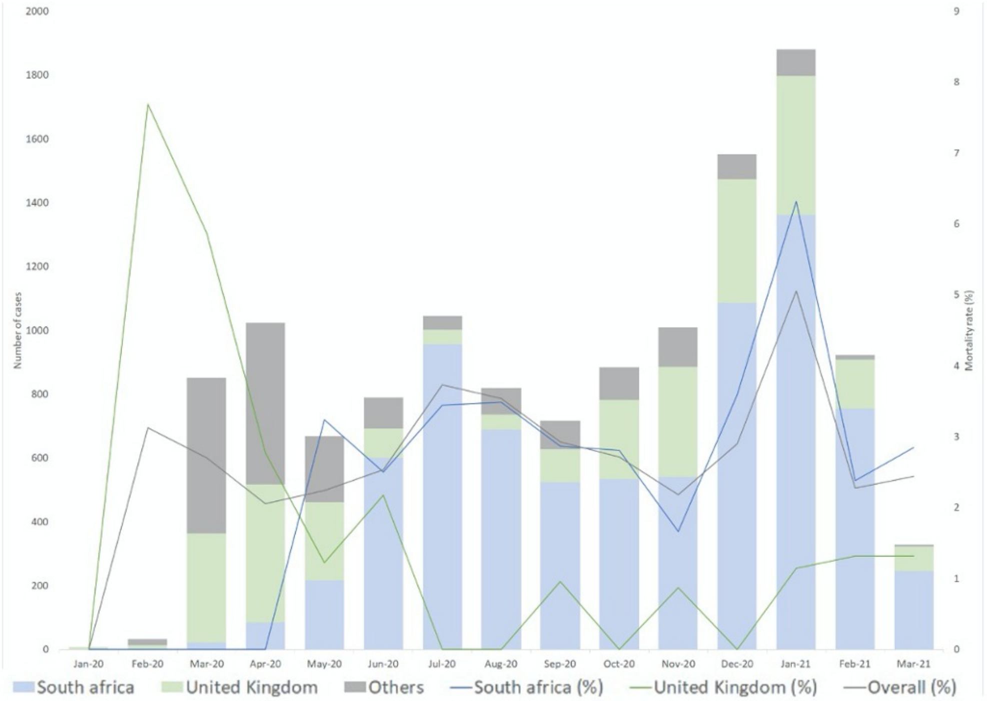

Figure 2. Total COVID-19 participants (bar) and mortality (line) rate trend throughout data collection period.

Figure 2. Total Covid-19 participants (bar) and mortality (line) rate trend through out data collection period.

363x270mm (72 x 72 DPI)

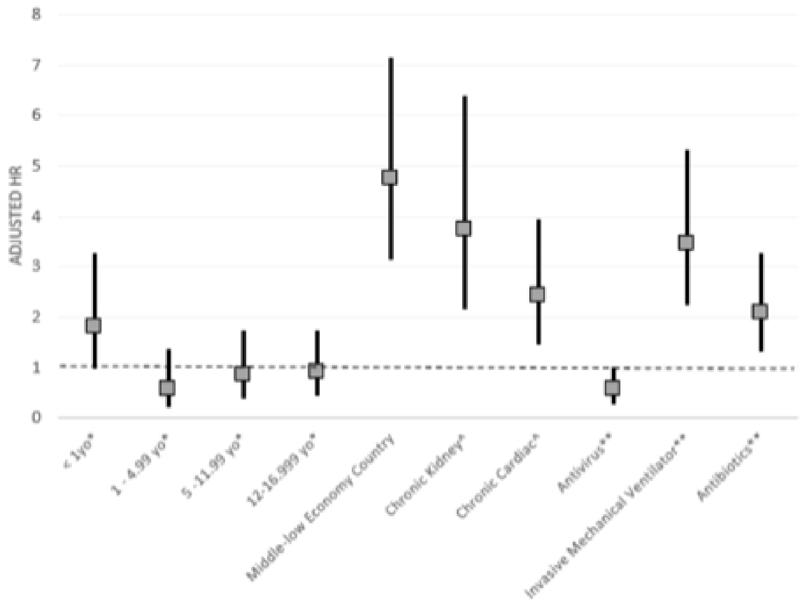

Figure 3. Adjusted Hazard Ratio and 95% Confidence Interval of mortality risk factors in all participants.

yo = years old; HR= Hazard Ratio

*) using age category ≥17 years as reference; ^) morbidity factors; **) COVID intervention factors

Adjusted Hazard Ratio and 95% Confidence Interval of mortality risk factors in all participants.
yo = years old; HR = Hazard Ratio
*Using age category >17 years as reference; ^) morbidity factors' **) COVID intervention factors.

96x81mm (150 x 150 DPI)
